# ^h^InGeTox: a human-based in vitro platform to evaluate lentivirus/host interactions that contribute to genotoxicity

**DOI:** 10.1038/s41434-025-00550-9

**Published:** 2025-07-15

**Authors:** Saqlain Suleman, Sharmin Alhaque, Andrew Guo, Huairen Zhang, Annette Payne, Marco Zahn, Serena Fawaz, Mohammad S. Khalifa, Susan Jobling, David Hay, Matteo Franco, Raffaele Fronza, Wei Wang, Olga Strobel-Freidekind, Annette Deichmann, Yasuhiro Takeuchi, Irene Gil-Farina, Jan Klapwijk, Stefany Perera, Manfred Schmidt, Michael Themis

**Affiliations:** 1 Department of Life Sciences, College of Health, Medicine & Life Sciences, Brunel University of London, Uxbridge, UK; 2 TestAVec Ltd, Queensgate House, Maidenhead, UK; 3 School of Life Sciences, Faculty of Science and Engineering, Anglia Ruskin University, Cambridge, UK; 4 Biomavericks, London, UK; 5 Department of Computer Science, College of Engineering Design and Physical Sciences, Brunel University of London, Uxbridge, UK; 6 Genewerk GmbH, Heidelberg, Germany; 7 Medical Faculty, University Heidelberg, Heidelberg, Germany; 8 Institute of Environment, Health and Societies, Brunel University of London, Uxbridge, UK; 9 Centre for Regenerative Medicine, The University of Edinburgh, Edinburgh, UK; 10 Division of Infection and Immunity, University College London, London, UK; 11 Division of Advanced Therapies, National Institute for Biological Standards and Control, Potters Bar, UK; 12 Cornelis Consulting Ltd., Hertfordshire, UK; 13 NCT and DKFZ, Department of Translational Oncology, Heidelberg, Germany; 14 Division of Ecology and Evolution, Department of Life Sciences, Imperial College London, London, UK

**Keywords:** Stem-cell differentiation, Molecular biology, Cell biology

## Abstract

Lentivirus vectors are effective for treatment of genetic disease. However, safety associated with vector related genotoxicity is of concern and currently available models are not reliably predictive of safety in humans. We have developed ^h^InGeTox as the first human in vitro platform that uses induced pluripotent stem cells and their hepatocyte like cell derivatives to better understand vector-host interactions that relate vectors to their potential genotoxicity. Using lentiviral vectors carrying the eGFP expression cassette under SFFV promoter activity, that only differ by their LTR and SIN configuration, we characterised vector host interactions potentially implicated in genotoxicity. To do this, lentiviral infected cells were subjected to an array of assays and data from these was used for multi-omics analyses of vector effects on cells at early and late harvest time points. Data on the integration sites of lentiviral vectors in cancer genes and differential expression levels of these genes, showed that both vector configurations are capable of activating cancer genes. Through IS tracking in bulk infected cell populations, we also saw an increase in the viral sequence count in cancer genes present over time which were differentially regulated. RNASeq also showed each vector had potential to generate fusion transcripts with the human genome suggestive of gene splicing or vector mediated readthrough from the internal SFFV promoter. Initially, after infection, both vector configurations were associated with differential expression of genes associated cytokine production, however, after culturing over time there were differences in differential expression in cells infected by each LV. This was marked in particular by the expression of genes involved in the response to DNA damage in cells transduced by the SIN vector, suggesting effects likely to prevent tumour development, in contrast to the expression of genes involved in methylation, characteristic of tumour development, in cells transduced by the LTR vector. Both sets of lentiviral infected cells were also found associated with differential expression of *MECOM* and *LMO2* genes known to be associated with clonal dominance, supporting their potential genotoxicity. Alignment of transcriptomic signatures from iPSC and HLC infected cultures with known cancer gene signatures showed the LTR vector with a higher cancer score than the SIN vector over time in iPSC and also in HLC, which further suggests higher genotoxic potential by the LTR configuration lentivirus. By application of ^h^InGeTox to cells infected with LV at the pre-clinical stage of development, we hope that ^h^InGeTox can act as a useful pre-clinical tool to identify lentivirus-host interactions that may be considered contributory to genotoxicity to improve safer lentiviral vector design for gene therapy.

## Introduction

Cell and gene therapy clinical trials have increased over the past 10 years from 1800 to 5000 listed with the National Institutes of Health that include new technologies involving CAR-T and gene edited cells. Currently, 40% of all trials are industry sponsored. In the US, each investigational new drug application (IND) requires FDA review, which primarily considers safety and may result in time consuming clinical holds on products entering the market. Recently, INDs have increased enormously with gene therapy product development and diversity and with commercial interest. Between Jan 2020 and Dec 2022, 33 clinical holds were announced that concerned CAR-T therapies (27%) and lentivirus vector-based therapies (15%) [1, 2].

Several gene therapy trials have successfully used gamma retrovirus (γ-RV) and lentivirus (LV) vectors for therapeutic gene delivery as they offer permanent gene transfer to the host genome after infection and integration. However, integration of the viral vector in the host genome risks insertional mutagenesis and differences in γ-RV and LV integration site (IS) selection are known to influence their genotoxic potential [3, 4]. Genotoxic effects by γ-RV includes changes in host protooncogene expression driven by the long terminal repeat (LTR) enhancer, as identified in ALD, X-SCID, WAS and CGD trials where oncogene upregulation resulted from vector integration in or near to the gene locus [5–9]. Promoter activation involving γ-RV integration is also known to cause gene upregulation [10]. A major improvement to reduce host gene activation upon integration was the development of LV vectors with the LTR modified to SIN configuration to abrogate its promoter and enhancer activity [11–13]. However, this modification has been reported to associate with readthrough from the internal promoter, used to replace the LTR function [14]. Regardless, SIN configuration LV that shows integration preference for the gene transcription unit, rather than the promoter region by γ-RV, coupled with 3^rd^ generation design to avoid the emergence of replication competence, has significantly improved the safety profile of these vectors. Concurrently, LV are considered highly suitable for the treatment of rare genetic diseases and for the generation of CAR-T cells for immunotherapy.

Unfortunately, genotoxicity still has been shown possible by LV vectors in certain configurations that can lead to oncogenesis following cancer gene activation [15, 16]. Further to this, LV splicing with host cancer genes can occur where novel gene fusions that are produced have the potential to drive clonal expansion [17, 18]. In a beta thalassaemia clinical trial, integration of a SIN LV resulted in 3’ end substitution of the *HMGA2* gene that abolished let7 microRNA control of this protooncogene, reduced *HMGA2* degradation and subsequent clonal proliferation [18]. In a clinical trial against chronic lymphocytic leukaemia, where LV was used to generate CAR-T cells for cancer immunotherapy, CAR-T cells were found to persist as a result of gene inactivation by the vector. In this case, intronic insertion resulted in removal of control of *TET2* after splicing with the LV vector. In a CD19 CAR-T trial, LV mediated oncogene activation has also been suspected to have caused CAR-T cell persistence [19, 20]. As a result of these findings, the FDA has determined that T cell malignancies are possible for all currently approved BCMA and CD19 autologous CAR-T immunotherapies and in turn all associated products are under investigation.

The role of epigenetics in cancer progression is well known [21, 22] and LV infection has been suggested to promote hepatocellular carcinoma development in mice. As a result of infection and the innate host immune response, protooncogene activation was shown to occur as a result of methylation changes to promoters under the control of the E2F transcription factor [23].

To understand RV and LV mediated genotoxicity more clearly and predict potential vector related side effects, in vitro and in vivo murine-based models have been developed. The in vitro immortalization (IVIM) assay that uses murine hematopoietic stem cells (HSC) has been shown useful for vector risk assessment. This model demonstrates differences in RV and LV IS preference and that integration in *Evi1* and *Prdm16* proto-oncogenes is responsible for cell transformation with RV risk being greater than LV by a factor of 3:1. Therefore, IVIM has been accepted by several regulatory agencies for pre-clinical safety evaluation of RV and LV. More recently data from this model has been used to provide transcriptomic signatures of leukemogenesis supporting its use as a surrogate assay for genotoxicity assessment (SAGA) [24]. In vivo models include *Cdkn2a* null mice with inactivated p53 and pRb pathways that have been valuable in showing that the risk of tumour development by LV vectors is approximately 10-fold lower than RV. In a fetal/neonatal murine model LV delivery has also been found associated with high frequency liver cancer [23]. Although the models currently used to understand and assess LV genotoxicity have proven valuable, they are still considered potentially biased or over sensitive relying on transformation. Furthermore, it is agreed that no test can reliably predict long term safety in humans with widely variable predisposition to cancer.

Recent concerns on gene therapy vector safety have led to a meeting of experts to discuss ways to define genotoxicity, uncertainty, suitable toxicity endpoints to use, and the need for future research with relevant models that predict safety prior to clinical gene therapy. As a result, a consensus opinion was reached and reported to support risk assessment of gene therapy products [25].

As a human based model is clearly urgently required, we chose to develop an alternative strategy for improving LV safety assessment, in which known genetic factors contributary to genotoxicity associated with LV/host interactions are firstly identified. For this, we chose human induced pluripotent stem cells (iPSC) and their hepatocyte-like cell (HLC) derivatives as these cells have been widely used to model human diseases and for pharmacotoxicological studies of disease treatment [26–28]. Patient iPSC also offer a personal approach to risk assessment by considering the genetic background of the host.

We have previously shown iPSC express markers of pluripotency and can be reliably reprogramed to HLC that are quiescent liver cells that match primary hepatocytes at the transcriptional level [29]. In this research report, we describe the development of the first Human Individualised Genotoxicity (^h^InGeTox) assay, as an in vitro platform, that uses iPSC and their HLC derivatives to understand in greater detail LV host interactions to provide further insight into oncogenesis potential and to use for improvement of LV safety design.

As retrovirus long terminal repeats (LTR) are known to be involved in cancer gene activation in the clinic and in non-clinical models, in development of ^h^InGeTox we used two LV that only differ by their LTR design, carrying native and SIN configuration LTR respectively, with the *eGFP* transgene under internal SFFV promoter activity. These were used to infect both iPSC and HLC, differentiated as spheroid cultures (hereafter denoted as 3D HLC). Data from vector/host interactions were subjected to multi-omics cancer associated analysis to characterise these interactions with the potential to support pre-malignancy and oncogenesis. Data from this analysis was also aligned to transcriptional signatures of a range of cancers to indicate genotoxicity potential by each LV configuration.

We propose ^h^InGetox as the first human-based model that offers detailed information on vector/host interactions that may be used to improve safe vector design to avoid genotoxicity and as a potential decision-making tool to support gene therapy LV product approval.

## Results

### iPSC and their 3D HLC derivatives express markers of pluripotency and liver-like cells, respectively, and are highly permissive to LV transduction

Bulk cultures of male JHU106i iPSC were differentiated to 3D HLC spheroids as previously described expressing markers of pluripotency and hepatocytes, respectively [29, 30]. Cultures were infected with 2nd generation HIV-1 based LV vectors carrying native LTR (pHV) or SIN LTR (pHR) configurations, respectively, and use an SFFV internal promoter to drive GFP expression (Fig. S1A, B). Flow cytometry was used to measure infection by GFP expression in iPSC at 90% and single cell dissipated 3D HLC at 85%, as previously described [31]. Normalised vector copy number (VCN), measured via TaqMan™ qPCR (*n* = 3 biological samples, each sample read in triplicate) on infected samples ranged between 1.5–9.0 VCN/cell. Significantly higher VCN was found in HLC infected with pHV compared to pHR, which contrasted the infection of iPSC (Fig. S1C).

### LV insertion site analysis

As integration in specific sites have been shown to contribute towards genotoxicity, insertion site (IS) analysis of LV was performed by Extension Primer Tag Selection Ligation-Mediated Polymerase Chain Reaction (EPTS/LM-PCR) [32] after infection. Biological replicates (*n* = 3) were used for each condition (i.e. timepoint and cell type). iPSC were harvested at two time points, 3 and 30 day post infection by LV whereas HLCs were harvested day 3 post infection for analysis due to their quiescent nature. In total, 412,786 integration sites (IS) were detected, with percentages normalised to the total number of IS across the genome. IS profiling of gene density, chromosome location, proximity to CpG islands and GC content and position within the gene transcription unit in both iPSC and HLC genomes were identified (data not shown) and appeared as previously reported for HIV-1 LV integration [3]. Locations within the transcription units was defined by regions transcribed into mRNA. Analysis of IS in non-cancer and cancer genes shows vector distribution in introns, 3’ untranslated regions (UTR), 5’ UTR and in exons (Fig. S2). To investigate the distance between IS and transcription start sites (TSS) in transcriptional units, the mean distance across each data set was plotted in the scale of 0–1 (exon or intron; normalised against gene length) or Log10 (3’ or 5’ UTR; value in base pairs). IS appeared evenly distributed in introns. IS identified in exons mainly congregated at 3’ end of genes (median insertion sites at 82.8% of averaged gene length). On average, inserts identified in 5’ UTRs were 27.9 kbp upstream of the ATG coding region and in 3’ UTR regions, inserts were 29.6 kbp downstream from the stop codon respectively.

For all genes, the IS number is highest in introns most likely because of size. There is a reduction in IS identified in iPSC cultures over the 30 day time period. This occurred for both vectors independent of LTR configuration and target site selection in the transcription unit (Fig. S2). A reduction in IS in non-cancer and cancer genes appears consistent between the two time points of 3 and 30 days after infection. Reduction in the number of IS found could be due to clonal selection or as a result of cell death due to insertions detrimental to cell survival. To examine for cell outgrowth, longer periods of culture would be necessary to study differences in IS selected by the vector that may influence cellular proliferation.

### LV IS associate with pathways representing cellular proliferative potential

**Fig. 1:**
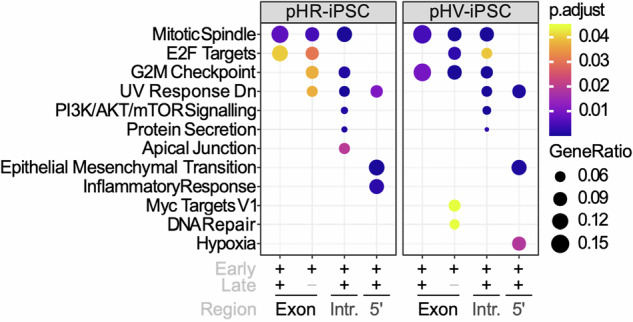
Pathway analysis of enriched hallmark gene sets in pHR or pHV infected iPSCs. Dot plots representing the most enriched signalling pathways within Hallmark gene sets for IS in pHR- or pHV-infected cells in early stage post infection or a combination of both across distinct regulatory regions, as denoted by + or −. Gene enrichment percentages are indicated by dot size. Benjamini–Hochberg adjusted *p* values are indicated by colour gradients (*p* < 0.05).

**Fig. 2:**
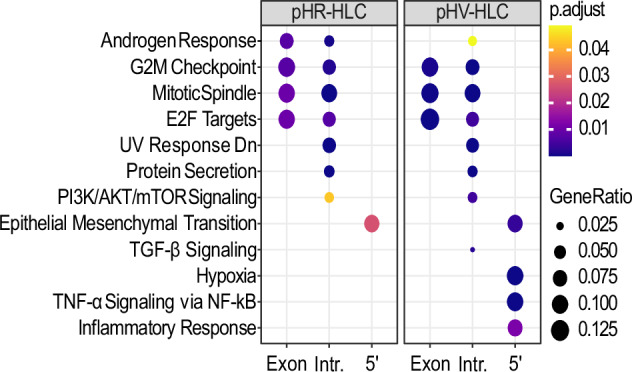
Pathway analysis showing enriched Hallmark gene sets in LV infected HLC. Dot plots of the enriched signalling pathways within Hallmark gene sets for insertion sites identified in pHR or pHV infected HLCs, harvested 3 days post infection, across distinct regulatory regions. Expression percentages of gene sets are indicated by dot sizes. The Benjamini–Hochberg adjusted *p* values are indicated by colour gradients (*p* < 0.05).

To gain insight into the enrichment of genes targeted by each LV involved in cancer pathways, hallmark pathway analysis was performed using the integration site analysis data from iPSC cultures obtained 3 days and 30 days post infection (Fig. 1). The level of enrichment is shown by dot sizes and colour intensity. Enrichment was found for IS in exons, introns, 5’ UTR but not 3’UTR regions. For both LV configurations, IS that were identified 3 days post infection enriched in exons for genes in significant pathways (*p* < 0.05) associated with cell cycle i.e. E2F targets, G2M checkpoint and DNA repair, were not identified at the later 30-day harvest time point. At the later time point, IS positioning in enriched genes appeared predominantly in introns and UTR regions of these genes and associated with the PI3K-AKT/MTOR pathway and pathways for epithelial mesenchymal transition. Between the two LV, differences in enrichment of genes were found for pathways involved in the inflammatory response (pHR) or hypoxia (pHV), respectively. Next, we looked into which genes were represented in each pathway. Interestingly, we found that most of the targeted genes were tumour suppressor genes. Compared to the pHR infected iPSCs, many of the tumour suppressor genes-associated inserts were identified in introns or UTRs in the pHV infected iPSCs at both early and late time points as opposed to these insertions being only present in early time point in pHR infected iPSCs. Targeted oncogenes were mainly identified in PI3K/AKT/mTOR signalling pathway. Compared to the infected iPSCs, HLCs exhibit enriched genes for the TNFα signalling pathway via NF-kB upon pHV infection with insertions in the 5’UTR and for the androgen response pathway in introns (Fig. 2). Fewer targeted genes associated with oncogenes or tumour suppressor genes was found in the infected HLCs. From these data, we concluded that both vectors are capable of insertion into genes important to oncogenesis and that the pHV with native LTR configuration appears to reside mainly in 5’UTR and introns over time where the LTR promoter may have greater influence on control of gene expression.

### Clonal tracking IS in iPSC exposes genes associated with clonal outgrowth

Since lentiviral insertion occurs in a semi-random manner, we looked at sequence count changes (SCC) in IS for all genes, regardless of positioning, using iPSCs between the early and late sampling timepoints. We used differential absolute SCC of ≥2-fold (Benjamini-Hoberg corrected *p* value *p* < 0.05) to represent only significantly enriched genes (*n* = 3 biological replicates) between the early and late assay timepoints. Differential analysis identified 717 targeted genes that included 23 oncogenes (10 pHR and 13 pHV) and 35 tumour suppressor genes (15 pHR and 20 pHV). Oncogenes and tumour suppressor genes that were identified in iPSC with SCC between early and late harvests were identified for both LV (Table S1).

The IS identified were always positioned in introns or UTR regions of genes. These were associated with eukaryotic translation, cell cycle regulation, kinases associated with protein phosphorylation and RNA export from nucleus. Once again, this identifies genes potentially involved in clonal outgrowth associated with insertions by both pHR or pHV LV.

### Analysis of gene expression changes in isolated iPSC clones infected by LV

To analyse directly the effect of LV integration on IS gene expression, single iPSC clones from pHR and pHV infections were isolated and expanded. DNA and RNA was then extracted for IS identification and qPCR analyses, respectively. Measurement of gene expression changes associated with each IS were compared to the expression of each respective gene in non-infected iPSC. For pHR and pHV, 27 and 29 IS genes, respectively, were identified significantly upregulated (> 2 fold) (Fig. S3). None of these genes were found to be downregulated, indicating positive vector influence on gene expression by both LV regardless of LTR configuration.

For further analysis of transcript identity beyond qPCR expression studies, we analysed the RNA Seq data derived from these isolated single cell iPSC clones. This identified a number of vector-host fusion transcripts. Of these transcripts, several were associated with LV insertion sites (Table S2). Mapping transcripts to each vector showed these to contain intron sequences suggesting gene splicing or readthrough may be the cause of altered gene expression. Further analysis of each fusion transcript is intended to identify regions common to each LV used for splicing events and quantify the ratio of splicing to readthrough by each LV.

### Differential expression of genes (DEG) in infected cells aligns with unique signatures representative of biological processes critical for oncogenesis

**Fig. 3:**
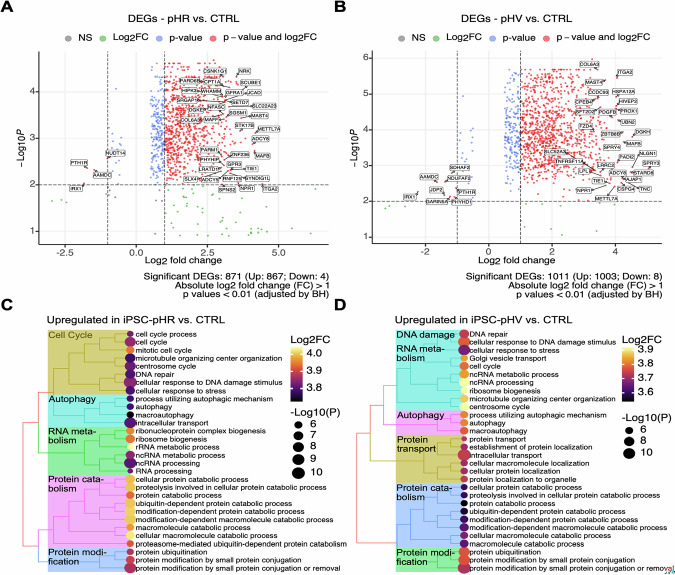
Investigation of gene expression changes in iPSC after lentivirus infection reveals mechanistic implications for oncogenesis. **A**, **B** Volcano plots illustrate genes that are upregulated (right, LogFC > 1) and downregulated (left, LogFC < −1) in infected iPSC, independent of sampling time, compared to uninfected controls. The volcano plot shows the most upregulated genes are on the right and the most downregulated genes are on the left. These genes are shown in boxes. **A** pHR and **B** pHV. Statistical significance is indicated in different colours. Highly significant genes have been labelled. **C**, **D** The top pathways with statistical significance and higher averaged expression levels of the genes characteristic of each pathway are shown. Dot plots reveal the main biological processes enriched in infected cells. **C** pHR and **D** pHV. Mean Log2 fold changes are indicated by colour gradients. The Benjamini–Hochberg adjusted *p* values for all graphs are indicated by dot sizes (*p* < 0.01).

To determine the global changes in gene expression upon infection by LV, RNASeq on infected bulk cultures was used to provide an unbiased transcriptome wide DEG profile against control uninfected cells. Compared to uninfected cells, when assessing infected iPSC (regardless of harvest timepoint), a total of 1011 DEG were associated with pHV with increases in 14 oncogenes and decreases in 14 tumour suppressor genes. Of 871 DEG associated with pHR, we identified increases in 10 oncogenes and decreases in 20 tumour suppressor genes. Those that were dysregulated by the greatest significance (*p*.adj < 0.01) and highest log2 fold change (absolute LogFC >1) are shown in red as upregulated and blue as downregulated in Fig. 3A, B. Common to both LV, GO term analysis of DEG identified annotated biological functions for signalling pathways that involve RNA transcription and protein modification. Between both vectors, common classes of signalling pathways were enriched including for autophagy, protein catabolism and protein modification, with unique classes enriched for each vector, including cell cycle (pHR) and DNA damage response (pHV) (Fig. 3C, D). Further, DEG were associated with strong immune signatures with cells displaying active cytokine production early after infection. In pHV-infected iPSCs, quite a few upregulated DEGs are implicated in methylation (*n* = 11; *NSUN7, PRDM15, SMYD4, CMTR2, CARNMT1, FAM86C2P, MTAP, METTL3, METTL15, METTL7A, TPMT*) and WNT signalling (*n* = 12; *LYPD6, PRDM15, SMURF2, ADGRA2, DAAM2, EDA, ROR2, RECK, SEMA5A, TTC21B, TGFB1, ZEB2*), partially characteristic of cancer development. In pHR-infected iPSCs, quite a few upregulated DEGs are implicated in response to DNA damage stimulus (*n* = 21; *BCL3, BCL6, CDKN2AIP, CTC1, DDX11, DCLRE1B, POLK, POLQ, FANCF, FAM111A, MICA, RAD52, SETD7, SLF1, SUV39H1, WRN, HROB, IRF7, MCM8, VAV3, ZC3H12A*) and activation of GTPase activity (*n* = 11; *GMIP, RUNDC1, TBCK, TBC1D2, TBC1D22B, USP6NL, WNT5A, NEDD9, SGSM1, SLC27A4, SYDE1*), partially suggestive of protection against cancer progression [33]. These data suggest LV with native LTR configuration associates with different cellular response to infection than SIN configuration LV suggesting greater genotoxic potential.

To determine whether the two differing LTR configurations are associated with DEG indicative of biological pathways towards cellular proliferation, DEG in cells infected by each LV configuration was compared between to control samples (Fig. 3). Compared to uninfected cells, pHR LV infected iPSC showed 419 DEG comprising 22 oncogenes and 10 tumour suppressor genes. These were implicated in tyrosine kinase receptor signalling and cellular senescence pathways. In contrast, 472 DEG were associated with pHV LV infected iPSC that comprised of 20 oncogenes and 13 tumour suppressor genes, representative of p13K and MAPK signalling pathway activation.

**Fig. 4:**
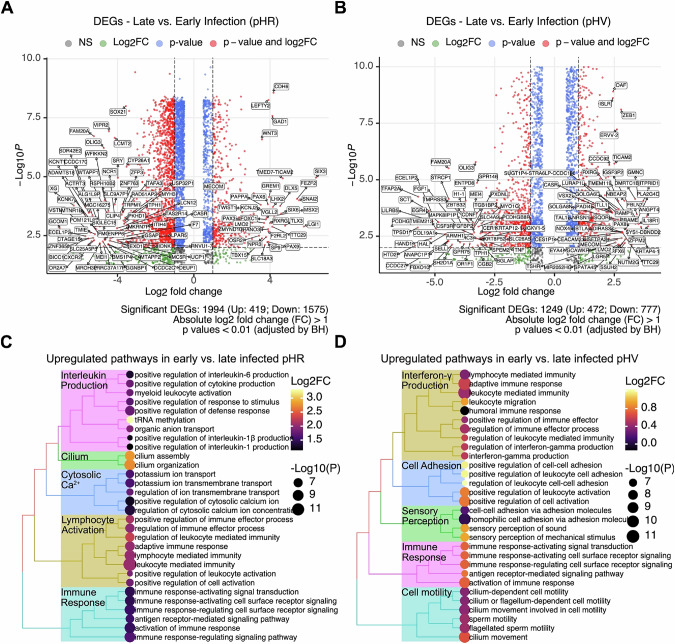
Investigation of gene expression changes in early vs. late harvested iPSCs post-lentivirus infection shows activated immune response with distinct pathway implications. **A**, **B** Volcano plots illustrate the upregulated (right, LogFC > 1) and downregulated (left, LogFC < −1) genes in pHR (**A**) and pHV-infected (**B**) iPSCs, comparing late to early harvest post-infection. Significance levels are represented by colour variations. Highly significant genes have been labelled. The volcano plot shows the most upregulated genes are on the right and the most downregulated genes are on the left. These genes are shown in boxes. **C**, **D** Dot plots reveal the main biological processes enriched in pHR and pHV infected iPSCs for both early and late harvests. In early harvested samples, both pHR and pHV infections show immune signatures, especially in cytokine production. Distinctly, late-harvested pHR-infected iPSCs predominantly show pathways related to DNA damage responses, while pHV infections are characterised by inflammatory pathways. For both LV configurations, DEG of *MECOM* and *LMO2* genes, known to be associated with clonal dominance were identified Mean Log2 fold changes are indicated by colour gradients. The Benjamini–Hochberg adjusted *p* values are indicated by dot sizes.

**Fig. 5:**
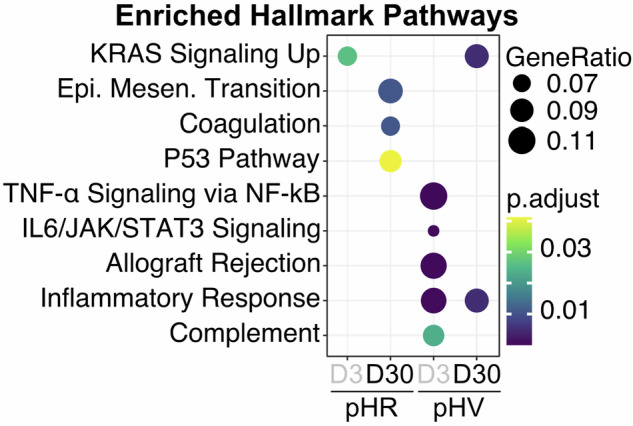
Pathway analysis reveals activated DNA damage response, EMT and inflammatory response in iPSCs post-lentivirus infection by Day 30. Dot plots illustrate enriched gene sets characteristic of Hallmark pathways between Day 3 and Day 30. Specifically, pHR-infected iPSCs at Day 30 are associated with genes characteristic of DNA damage response, EMT and coagulation pathways, while pHV-infected iPSCs are associated with inflammatory response. Expression percentages of gene sets are indicated by dot sizes. The Benjamini–Hochberg adjusted *p* values are indicated by colour gradients.

When comparing DEG in infected iPSC at the early stage for both LV, these cells exhibit active cytokine production (Fig. 4C, D). To compare DEG between the early and later time points cells, we used volcano plots to show the most upregulated and downregulated genes. These are shown for pHR and pHV where the genes on the right are most upregulated and the most downregulated genes are shown on the left, presented in boxes (Fig. 4A, B). For both LV configurations, DEG of *MECOM* and *LMO2* genes, known to be associated with clonal dominance, were also identified. Biological pathways for DEG are presented in Fig. 4C, D. Dot plots illustrating enriched gene sets characteristic of Hallmark pathways between early and later time points are shown where the major difference between pHR and pHV infected iPSCs is characterised by DEG corresponding to the p53 pathway responding to DNA damage in pHR infected cells versus DEG corresponding to the inflammatory response in pHV infected iPSCs (Fig. 5). These results confirm differences in global gene expression involving cancer genes occurs upon LV infection by either LTR configuration.

HLC infected by each vector was harvested only at the early 3 day time point and DEG were identified and compared with uninfected cells. Analysis in HLC infected by pHR LV found 569 DEG of which 37 were oncogenes and 51 tumour suppressor genes. In contrast, infection by pHV LV resulted in identification of 3762 (seven-fold increase) DEG of which 81 oncogenes and 82 tumour suppressor genes, that included *MECOM*, *LMO-2* and *BRAF* genes previously associated with genotoxicity [8, 15, 24]. These were not present in the pHR DEG suggesting stronger genotoxic potential by the native LTR configuration vector. Chi square test analysis reveals no significant difference between the number of oncogenes and tumour suppressor genes differentially expressed by pHR or pHV LV in HLC (Chi-Square = 1.0523, *p* value = 0.305). GO term analysis of these data showed genes mainly associated with tyrosine kinase signalling (*n* = 23 genes) and protein phosphorylation (*n* = 19 genes) and their related pathways, such as ERK1/2 cascade and PI3K/AKT/mTOR pathway. For pHV infected cells, GO term analysis of the upregulated DEG also identified pathways involving chemotaxis (*n* = 23 genes) and cancer signalling pathways (*n* = 36 genes), including NF-kB (*n* = 69 genes), MAPK (*n* = 54 genes), Wnt (*n* = 39 genes), JNK (*n* = 38 genes) and PI3K/AKT. In contrast, pHR infected cells show pathways characterised by groups of genes protective against viral infection such as interferon-associated genes (*n* = 5), DNA damage (*n* = 35 genes), p53-mediated apoptotic proteins (*n* = 3), zinc finger proteins (*n* = 3) and autophagy (*n* = 25). These results indicate the oncogenic shift in gene expression after gene transfer appears greater for the native LTR vector.

### Weighted gene co-expression network analysis (WGCNA) reveals distinct LV associated gene expression

**Fig. 6:**
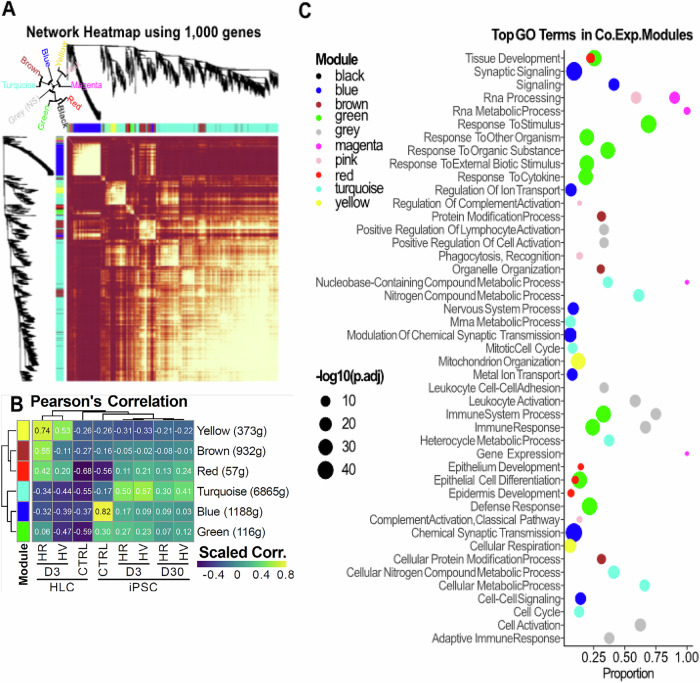
Identification and characterisation of co-expression modules through weighted correlation network analysis (WGCNA). **A** Transcriptomic data from 1000 genes from all LV infected samples were analysed through WGCNA and grouped into common co-expression modules. The heatmap shows the main co-expression modules which were identified, each representing different biological functions. **B** 6 co-expression modules were found to be highly significant across all LV infected samples. A heatmap of these modules are shown for each sample using Pearson’s correlation. This highlights differences between each sample, with an association generally shown after LV infection regardless of vector configuration. The brown, turquoise, and green modules particularly have a significant overlap of genes, including those preventative of tumourigenesis. **C** Dot plots showing the main biological implications of each co-expression module in protein modification (brown), cellular metabolism (turquoise), synaptic signalling (blue), stimulus and immune response (green), epithelial cell differentiation (red), RNA metabolism (magenta), phagocytosis (pink) and cellular respiration (yellow) indicating crossover in biological processes targeting by the vector for IS preference. The Benjamini–Hochberg adjusted *p* values in −Log10 are indicated by dot sizes.

WGCNA is a systems biology approach used to identify transcriptome-wide relationships of all genes rather than individual genes in isolation. Genes with similar expression patterns, that may be up or downregulated in their expression or belong to pathways with similar functionality, are placed into modules. Genes that are expressed and belong to these modules may also be analysed for enrichment. Hence, to better understand biological processes that may be influenced by LV infection, we profiled unique gene expression programmes across iPSCs or HLCs particular to each LV using transcriptomic data for this unbiased approached. Firstly, RNASeq was used to identify the expression of 1000 genes from the LV infected samples that could be grouped as co-expressed genes (Fig. 6A). Those with high significance were then placed into co-expression modules. These modules are indicated by colour codes, gene numbers (g), and percentage of shared inserted target genes (Table S3). We found that modules coloured in Brown, Turquoise, and Green are associated with higher proportions of shared inserted target genes and tumour suppressor genes (TSG). These modules were further found to be associated with pHR and pHV infected iPSC or HLC using Pearson’s correlation (Fig. 6B). The functional implications of each module were assessed and shown as dot blots (Fig. 6C). We found that these modules are associated with biological processes critical for protein modification (Brown), cellular metabolism (Turquoise), synaptic signalling (Blue), stimulus and immune response (Green), epithelial cell differentiation (Red), RNA metabolism (Magenta), phagocytosis (Pink), cellular respiration (Yellow). High scores indicate high similarity, whilst low scores indicate little correlation.

For pHR infection, protein modification, cellular metabolism and stimulus and immune response were associated with high proportions of shared genes including tumour suppressor genes, although in general there is a shift to an increased association with these modules after infection regardless of the LV used (Fig. 6B). A greater shift of association occurred in infected HLCs compared to iPSCs where there is a small decrease in association over time. Interestingly, there is a greater shift of association with these modules after pHR infection compared to pHV.

These data show that each LV is clearly associated with dysregulation of critical biological processes that are known implicated in oncogenesis [34–39], however it is difficult to quantify the contribution of these changes to genotoxicity.

### Gene splicing with LV and the human genome in infected cells

**Fig. 7:**
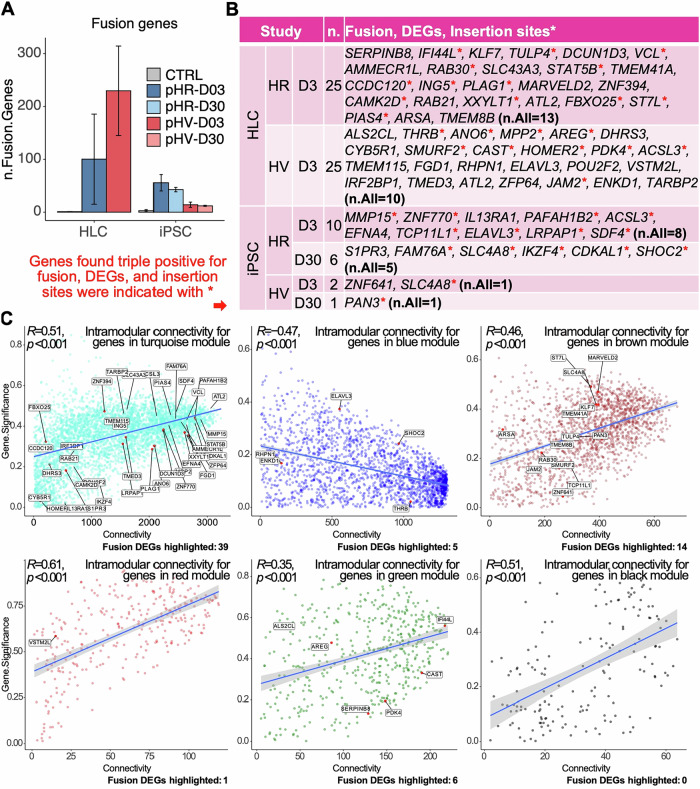
Analysis of fusion genes highlights their role in differential gene expression and lentiviral insertion patterns associated with key biological processes. **A** Bar charts showing the total number of fusion genes in pHR/pHV-infected iPSCs or HLCs harvested on Day 3 or 30 before analysis for vector/host fusions. SEM shown between three replicates analysed. **B** Table summarising specific fusion genes containing vector and host sequences identified for each treatment, with asterisks indicating those genes also differentially expressed and associated with insertion sites. **C** Scatter plots highlighting the significance of fusion genes within each co-expression module. Genes with high connectivity (i.e., central genes) are pivotal to the module biological function, compared to those with less connectivity (i.e., peripheral genes). Vector host fusion genes identified are predominantly present in the turquoise (cellular metabolism), brown (protein modification), and green (immune response) modules (n = gene number).

Gene splicing and readthrough is known to occur between LV and the targeted host genome and aberrant splicing is also known to cause changes in cancer gene expression [40]. Once again RNASeq of total RNA transcripts was used to identify novel fusion transcripts. We identified a total of 763 fusion genes across all iPSC and HLC infected cells involving both vectors of which 69 contained both vector and host sequences (Fig. 7A). The majority of these fusions showed vector integration within intron gene regions. Mapping of these fusions back to the vector genome is intended to determine common sites used by the vector for splicing with host genes and quantify the ratio of splicing to readthrough associated with each LV.

Of these DEG for both LV, 38 genes were also found as fusions and represented as IS (Fig. 7B). These triple positives (fusions genes, IS and DEG) were present as a smaller proportion in infected HLC (46%) compared to iPSC (79%), most likely due to higher gene expression in iPSC. Once again, the fusion genes identified were associated with cellular metabolism (*n* = 39), protein modification (*n* = 14), and stimulus and immune response (*n* = 6) module categories (Fig. 7C). Whereas the majority of pHR genes are associated with pathways involving the immune response, those associated with pHV are predominantly involved in metabolism with several highly relevant to cancer, suggesting this vector to have higher genotoxic potential.

### Transcriptome changes that align with cancer-specific gene signatures suggest trends in LV associated genotoxicity

**Fig. 8:**
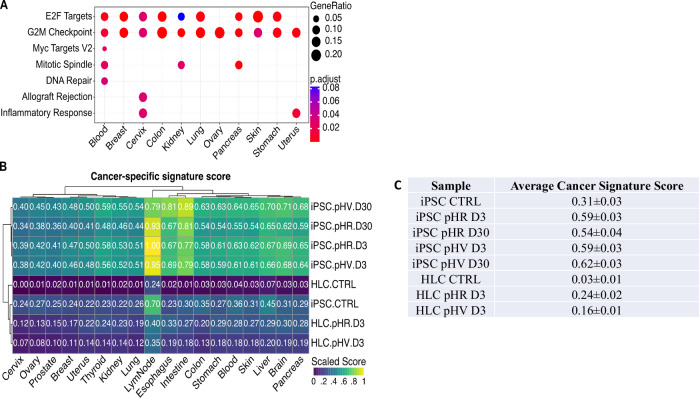
Evaluation of the potential carcinogenic impact of pHR or pHV lentiviral infection. **A** Dot plots of the most significantly enriched signalling pathways within Hallmark gene sets for cancer specific molecular signatures. Expression percentages of gene sets are indicated by dot sizes. The majority of these pathways are shown to be significant (*p*.adjust < 0.05). **B** Heatmap illustrating the cancer-specific scores for uninfected (control) and infected iPSCs and HLCs. **C** Average cancer signature scores, shown with SEM, to identify trends in data. Overall, a shift towards oncogenic gene signatures is demonstrated subsequent to LV infection. Notably, iPSCs generally exhibit higher cancer propensity scores than HLCs, potentially due to their higher proliferation. At Day 3 post-infection, pHR infected cells demonstrate elevated cancer-specific scores relative to pHV infected cells, consistent with earlier observations. By Day 30, however, pHV infected iPSCs exhibit higher scores than their pHR infected counterparts, suggestive of carcinogenesis.

To profile LV host interactions with carcinogenesis probability, we firstly defined highly relevant cancer-specific signatures to several cancer types using differential gene expression analysis compared to their normal tissues before LV infection. Through pathway analysis, using GO terms or hallmark gene sets, we found these signatures associated with enriched pathways involving nucleic acid synthesis/metabolism, active transcription, cell proliferation, E2F targets and the G2M checkpoint (Fig. 8A). These significant signatures were then used to score against the transcriptomes of infected iPSC or HLC from early or late harvest data analysis (*p* > 0.05, Fig. 8B). The average overall cancer signature score across all cancer profiles highlights differences between samples (Fig. 8C). Importantly, the cancer signature scores provide initial insights into vector-associated genotoxicity by indicating exploratory trends rather than statistically significant differences.

In general, infected iPSCs are characterised by higher cancer scores than infected HLC as expected due to their proliferative status. At the early harvest time point, pHR infected iPSC or HLC have higher cancer scores than pHV infected cells, in agreement with the IS and DEGs we identified earlier. However, after continued iPSCs culturing, sample harvesting at day 30 clearly associated pHV infected cells with higher cancer scores than pHR late cultures, revealing possible differences in the genotoxicity potential of the pHV vector (Fig. 8B). Focussing on liver cancer using HLC data, scores were different for the association between HLC infected with pHV and pHR at 0.2 and 0.29, respectively, compared to HLC CTRL (uninfected control) at 0.07. Further validation in future studies with established control vectors would be required to provide reliable genotoxic significance to compare vector genotoxicity potential.

### Epigenetic analysis reveals unique lentivirus-induced methylation profiles

DNA methylation is known to be associated with cancer [23]. We investigated whether epigenetic changes, in the form of DNA methylation could be used as an indicator of genotoxic potential for each LV. To do this, we analysed differentially methylated regions (DMR) in iPSC harvested at the late time point after LV infection. Hyper or hypomethylation of regions of regulatory elements were quantified and indicated using different colour codes in Fig. S4A. iPSC infected with pHV LV were characterised with increased hyper and hypo methylated regions compared with the pHR vector. The numbers of differentially methylated CpG island together with other regulatory elements including open-sea, shelf, and shore were found to peak at the gene body and reduce elsewhere. These remained higher in hypermethylated regions than hypomethylated regions. This trend was similar in pHR infected iPSC and HLC, suggesting both LV capable to induce general hypermethylation.

By focussing on CpG island hyper or hypomethylated promoter regions (TSS1500 or TSS200), there was a marked increase in the number of hypermethylated genes corresponding to pHV infection of iPSC (*n* = 210) than pHR infected iPSC (*n* = 24) or HLC (*n* = 28) (Fig. S4B, C). Through pathway analysis, we found hypermethylated signalling molecules associated with intracellular signal transduction enriched in these pHV infected iPSC (Fig. S4D). Several pathways, such as regulation of neurotransmitter levels and chemical synaptic transmission, known to be associated with cancer genes were found hypermethylated in pHV infected iPSC. Collectively, these data clearly suggest once again pHV with higher potential for genotoxicity than pHR.

### Multi-omics analysis shows pathways associated with cancer-related genes are shared between assay data sets

**Fig. 9:**
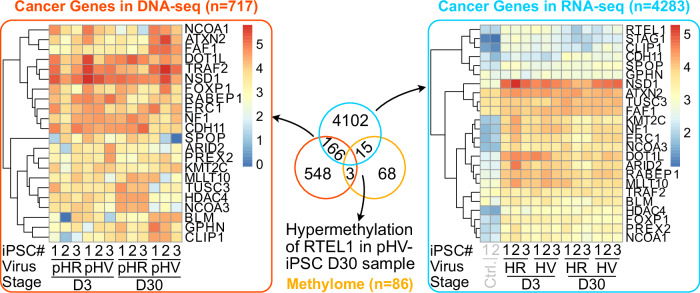
Omics data analysis between cancer genes commonly shared between DNA-seq (IS), RNA-seq and methylation data sets. Gene numbers are shown in a logarithmic scale. A delta beta value of greater or less than 0.2 was used to indicate hyper or hypo-methylation of each methylated region, respectively, and quantified the number of counts in log2 across each sample with regulatory elements. This highlights genes that have been identified as IS, differentially expressed and differentially methylated to identify trends between analyses. While no gene shares characteristics between all three data sets, common trends are identified between two data sets, highlighting the genotoxic potential of genes.

We next investigated, using assay data sets for both LV, whether cancer genes with IS could also be found with altered DMR and DEG in LV infected iPSC (Fig. 9). No genes were found overlapping in data sets for all three, however, some genes for cancer pathways were shared for LVs: methylomic and genomic (*n* = 3); methylomic and transcriptomic (*n* = 15) and genomic and transcriptomic (*n* = 166). Between the different data sets, pathways were enriched concerning cell signalling and associated with cell adhesion. Specifically, to methylomic and transcriptomic data, pathways were enriched for DNA repair of double strand breaks and for transcriptomic and IS data, enrichment pathways for apoptosis were observed. Closer examination of these pathways revealed them prominently to involve tumour suppressor genes important to carcinogenesis.

To assign these pathways to each vector as a measure of genotoxicity, GO term or KEGG enrichment analysis on these tumour suppressor genes in IS data (*n* = 717) identified DNA repair pathways important to DNA damage mainly enriched in pHR infected iPSC in contrast to pHV iPSC or HLC infected cells. In pHV infected iPSC, tumour suppressor genes were enriched that were characteristic of positive regulation of autophagy (Day 3) and negative regulation of cell proliferation (Day 30), suggesting a protective role against carcinogenesis. Importantly, hepatocellular carcinoma-associated tumour suppressor gene pathways were found enriched only in pHV infected HLC only, again indicating genotoxic potential for this vector (Fig. S5).

## Discussion

In this report, we developed ^h^InGetox to use as a pre-clinical tool to identify interactions between two LV vectors of similar architecture, differing only by their LTR configuration, with the human genome to identify factors considered with potential genotoxic and contributory to oncogenesis. The information gained from this study is intended to support improvement in LV design for safer gene therapy and meet some of the requirements of a consensus agreed amongst leaders in the field for risk assessment of gene therapy products [25]. It also aims to avoid bias in evaluating vector safety associated with the currently used models of risk assessment.

^h^InGetox uses a modular approach to identify vector/host interaction, which include vector integration site (IS) selection in host cancer genes with concomitant effects on their expression, global changes to differential expression of genes (DEG) and pathways associated with these changes. It also identifies vector/gene truncations that generate novel fusion transcripts suspected to alter cancer gene expression as has been described for a number of cancers [18, 40, 41]. Lastly, we used ^h^InGetox for identification and analysis of epigenetic modifications in form of methylation changes in the host genome that have the potential to change cancer gene expression and potentially drive oncogenesis [23, 42].

To develop ^h^InGetox, LV were used that carry either the native HIV-1 long terminal repeat (LTR) sequences or a self-inactivating (SIN) LTR configuration, which was developed to abrogate promoter/enhancer activation of cancer genes by the virus LTR [11]. While these both contain an internal strong SFFV promoter, we felt it important to focus our investigate on interactions between vector and host with different LTR configuration as the LTR has been shown to influence of oncogenesis [13, 15]. To avoid the use of immortal cell lines with existing mutated cancer gene pathways or mice, known to be highly sensitive to LV mediated oncogenicity, we chose for our analysis human induced pluripotent stem cells (iPSC) reprogrammed to hepatocyte-like cell (HLC) 3D organoid derivatives as surrogate hepatocytes. iPSC offer a highly proliferative state favouring LV integration, whereas HLC are quiescent. iPSC and HCL presented markers of pluripotency and of hepatocytes, respectively, as previously described [29]. The liver was used as the model of choice because it has been used for several drug and pharmacotoxicological kinetic studies [30]. Both iPSC and HLC were highly permissive to infection with high cell survival and viability matching that of untreated cells, favourable to for ^h^InGetox investigation. Although all infected cells were positive for GFP expression, levels of expression by each promoter were not measured in this study.

A total of 412,786 LV integrations were characterised in total in regulatory regions, revealing both LV configurations distributed in transcription units in the order of introns > UTR regions > exons. Hallmark pathway analysis of IS genes presented several similar associated pathways for both LV. These included E2F transcription factor targets in oncogenes, G2M checkpoints and DNA damage and repair and pathways of the inflammatory response enriched. We noticed, however, that sequence counts reduced for IS over time in iPSC. HLC were not examined for sequence count changes over time. Although the profile of IS remained the same for non-cancer and cancer genes, cancer gene IS became more enriched, suggesting possible clonal selection or cell death due to insertions in genes detrimental to cell survival.

Interestingly, IS found at the early time point (3 day) for both LV appeared in exon, introns and 5’UTR regions in genes associated with E2F targets, G2M checkpoint and DNA damage. Following clonal tracking, however, at the late time point, particular to the native LTR vector, insertions remained in cancer genes predominantly in introns or UTR sequences. Hallmark analysis showed these to be involved in pathways associated with the PI3K-AKT/MTOR pathway, epithelial mesenchymal transition, eukaryotic translation, cell cycle regulation, kinases associated with protein phosphorylation and RNA export from nucleus. Most importantly, targeted genes found significantly enriched in each pathway were tumour suppressor genes. This possible enrichment may favour cellular outgrowth caused by the LV LTR. Interestingly, IS were also identified in *SET*, *BRAF* and *MECOM* that have previously been found associated with genotoxicity studies [15, 24]. Since iPSC can be grown indefinitely and we have found HLC can been cultured for over 700 days in vitro, to examine for potential iPSc or HLC outgrowth, we intend further extended culture of cells after LV exposure.

Modified transcripts generated as gene fusions arise most likely from vector/host gene splicing or readthrough from the internal promoter into host genes past the vector 3’ polyadenylation sequences. Gene splicing between LV and host genes has been shown to cause premature termination of gene transcripts, thereby altering gene expression levels of genes and haploinsufficiency. In genotoxicity models and in a β-thalassaemia trial, aberrant splicing eliminated control of *HMGA2* gene transcripts causing gene upregulation and clonal expansion has been reported [18, 40, 41]. CAR-T cells have also been found to persist due to loss of control of proto-oncogene expression [19, 20] and several clinical holds on CAR-T therapies have been imposed by regulators.

Interestingly, fusions identified by RNASeq in this study appeared more prominently in SIN LV infected cells and greater in differentiated cells compared to pluripotent cells (> 2 fold). These fusions were mainly associated with protein modification, synaptic signalling and immune response and contained mainly host intron sequences suggesting they had arisen either from readthrough from the internal promoter of the vector, as has been previously described [43], or splicing between vector and host genome. Of the total 69 fusions identified for both LV from bulk cultures, 38 were found in common with DEG and IS, suggesting both LV capable of genotoxicity. However, the greater number of fusions identified with the SIN LV over time indicates the potential mechanism of genotoxicity by this vector may differ from the wild type LTR LV. Importantly, most fusions identified with the SIN LV were found for genes associated with pathways involving the immune response, whereas those associated with the native LTR vector were mostly with genes involved in metabolism with several highly relevant to cancer, suggesting this vector to have higher genotoxic potential.

Analysis of iPSC clones isolated following infection by each LV revealed only activation of genes (> 2 fold) local to insertion using quantitative PCR indicating both LV have genotoxic potential. Since SIN LTR configuration is believed not to be able to drive gene activation, we suspected this due either to readthrough out of the vector driven by the strong internal SFFV promoter, inactivation of gene control following integration as reported for control of the Tet 2 gene during CAR-T therapy [19], alternative gene splicing with host genes where novel gene fusions that drive clonal expansion [17, 18] or loss of control of gene expression as shown in a beta thalassaemia clinical trial [18, 44]. Interestingly, in the clones isolated from LV infection, gene fusions involving the vector, mainly in introns of activated genes, appeared at least once in every clone, suggesting truncation may play an important part in genotoxicity regardless of LTR design. Further analysis of the regions used by the vectors in this process is underway to identify common regions that could be altered to reduce the production of modified cancer gene transcripts.

To determine global effects on transcription in cells infected by each LV, RNASeq of total RNA was used to identify changes of DEG globally. GO analysis of genes in iPSC early after infection by each LV configuration revealed common biological functions for signalling pathways associated with strong immune signatures for active cytokine production. However, after culturing cells further, for pHV infected cells, DEG involving in genes implicated in methylation were identified characteristic of cancer, whereas in contrast in pHR infected cells, DEG were found for genes that were associated with DNA damage, which is indicative of protective against cancer. This once again suggests pHV with greater genotoxic potential. Importantly, at both early and late time points, pHR LV associated with DEG that were implicated in tyrosine kinase receptor signalling pathways and cellular senescence in contrast to pHV LV where DEG associated with p13K and MAPK cancer signalling pathway activation. Interestingly, for both LV configurations, DEG were also identified for *MECOM* and *LMO2* genes that have previously been found associated with clonal dominance.

In HLC, DEG associated with the LTR vector were 7-fold that of the SIN vector, with upregulation of several oncogenes and tumour suppressor genes and pathways mainly associated with tyrosine kinase signalling, protein phosphorylation and other ERK1/2 cascade and P13K/AKT/mTOR related pathways. While this may be in part due to the 2-fold higher VCN in pHV infected HLCs, DEG were again evident for *MECOM*, *LMO-2* and *BRAF* genes. Using GO term analysis, the upregulated DEG associated with pHV were identified to be involved in pathways for chemotaxis and cancer signalling, including NF-kB, MAPK, Wnt, JNK and PI3K/AKT. This was in contrast to DEG in pHR infected cells where pathways involving genes protective against viral infection were found, demonstrating once again the vector with LTR configuration may have a higher genotoxic potential, particularly in terminally differentiated cells.

To understand biological processes influenced by LV infection, unique gene expression programmes across iPSCs or HLCs particular to each LV using transcriptomic data were profiled for unbiased weighted correlation network analysis (WGCNA). The nine modules identified were cellular respiration, protein modification, epithelial cell differentiation, cellular metabolism, synaptic signalling and stimulus and immune response and Pearson’s correlation provided a statistical measure of the similarity between sample gene expression and biological process with high scores indicative of high similarity. For pHR more than pHV infection, protein modification, cellular metabolism and immune response were associated with high proportions of shared IS and these predominated in tumour suppressor genes. Although each LV appeared associated with dysregulation of these processes, that are known to be implicated in oncogenesis [34–39], it is difficult to accurately assign cancer risk to vector configuration.

Following virus infection, methylation is believed to be an innate mechanism used to prevent successful virus establishment and propagation [45]. We hypothesised that this innate immune response to virus infection may also cause methylation of host genes and carries the potential for further vector mediated genotoxicity. Hence, we profiled CpG methylation in iPSC and HLC genomes for specific genes and crossed referencing these with IS and DEG data to identify consensus cancer genes and their ontologies. Overall, LTR vector infected iPSC were 10 times more hypermethylated than SIN LV infected cells, with enrichment of genes involved in signal transduction associated with cellular proliferation indicating the LTR has a major influence on the epigenetic response to infection. This has also been shown in mice following infection by LV, where changes to methylation profiles altered the expression of cancer genes under the control of the E2F transcription factor [23].

To combine the data on factors potentially contributory to genotoxicity for each LV, multi-omics was applied to vector IS and cellular DEG and differential methylomics data. Whilst none of the genes for each LV were identified in all three data sets, data from suspected genotoxic factors were found overlapping with several sharing cell signalling pathways regarding cell adhesion. Distinct pathways between transcription and methylomic data sets were found for genes involved in DNA damage response and transcription and genomic sets for genes involved in the apoptotic response, respectively.

Tumour suppressor genes can provide an indication of early events leading to cancer, since altered sequence, methylated state, and or expression levels of these genes are thought to be implicated in the carcinogenic process. When performing GO term or KEGG enrichment analysis on these genes in the genomic data, we found that DNA repair pathways were mainly enriched in SIN LV infected iPSC compared to LTR vector infected iPSC or HLC. Conversely, in LTR LV infected iPSC, genes characteristic of positive regulation of autophagy (early time point analysis) or negative regulation of cell proliferation (late time point analysis) were found enriched, suggestive of a protective role against carcinogenesis, possibly being driven by the vector. Interestingly, hepatocellular carcinoma-associated tumour suppressor genes were found to be enriched in LTR vector infected HLC. Pathway analysis for tumour suppressor genes in transcriptomic data showed a number of intracellular signalling pathways were upregulated in infected iPSC by both LV and only a few signalling pathways like p53 were identified across different conditions in HLC.

In an attempt to assign carcinogenesis potential to each LV LTR configuration, cancer signatures associated with enriched pathways of several cancers were used for alignment with signatures scored from the transcriptomes of infected iPSC or HLC (early and late time points). Our finding that infected iPSC are characterised with higher associated cancer scores than infected HLC is most likely due to the nature of transcription in iPSC representing rapidly proliferating cells compared to differentiated HLC. In agreement to our observation for IS and DEG that we identified at the early time point analysis after infection, SIN LV infected iPSC or HLC had higher cancer signature scores than LTR vector infected cells. However, following extended culture, higher cancer scores became associated with the LTR vector suggesting transcription of cells with particular IS developing oncogenic expression profiles and revealing the genotoxic potential of the LTR vector. Cancer signatures, particularly with higher similarity with lymph-node signatures than liver cancer signatures, were believed due to the role of the immune response in carcinogenesis as active immune responses often play a protective role against cancer development and the fact that tumorigenesis is associated with immunosuppression [46]. Here, the identification of immune transcripts likely contributes to the prominence of the lymph-node signatures we observed. Lymph nodes are also key sites for immune cell activation and proliferation, and hence why enrichment of these signatures was evident in our data. Additionally, the liver has a unique immune environment that is often more tolerogenic [47]. This may result in a less pronounced immune response signature compared to lymph nodes, which are more actively involved in immunity.

Our modular analyses clearly show differences between LV configurations for genotoxic potential. Although we consider these findings go some way to being useful to identify factors carried by the vector that contribute to oncogenesis, it still remains difficult to predict cancer development in humans with long lifespan. Important to future work, hInGetox should be applied to LV with biologically relevant weaker or tissue specific promoters used clinically to identify genotoxicity outreads. Vectors used previously in clinical trials where leukemogenesis have been identified should also be tested.

In conclusion, we find that both native LTR and SIN configuration LV carry genotoxic risk by being able to integrate into and alter the expression of cancer genes. Insertions were also found in *MECOM*, *LMO-2* and *BRAF* that are known to be implicated in clonal outgrowth. Also, of importance is our finding that fusion genes can be generated by both LTR and SIN configuration and changes to methylation profiles of regions under the control transcription factors cause by LV also risks changes to the expression of cancer genes. Potential for oncogenic progression was also highlighted by alignment of the transcriptome of infected cells with cancer transcriptome signatures and infers the SIN LTR vector is less genotoxic than the native LTR carrying vector long term.

As murine-based models are believed unreliable to predict oncogenesis in humans and limited data can be obtained through clinical observations, it is hoped that ^h^InGetox provides an alternative. Rather than using unpredictable models for transformation or tumorigenesis as a genotoxic outread, ^h^InGetox offers a way to reduce genotoxic risk by testing the vector design for interactions with the host that may be considered contributory to genotoxicity and to use this information to modify the vector to reduce these interactions. Examples of changes that improve vector safety could be codon optimisation of the vector, reducing CpG content or removal of splice donor or acceptor sites in the vector. Following this, ^h^InGetox may be used to re-evaluate the new vector design. Because human iPSC reprogramming is possible to several cell types, we are currently developing ^h^InGetox in T cells intended as a model to investigate vector interactions in CAR-T cells. ^h^InGetox is also currently being transferred to screen AAV host interaction. It is hoped that ^h^InGetox will generate data that enables AI through in silico risk assessment of vector design as a platform for decision-making that supports regulatory approval of safe gene therapy products.

## Online methods

### Growth and characterisation iPSC pluripotency and differentiation to hepatocyte-like cells

A human iPSC line (JHUP106i) was cultured routinely on laminin 521 (BioLamina, France) coated plates in serum-free mTeSR™1 medium (STEMCELL Technologies, Cambridge) as previously described [48]. The cell was monitored regularly for infection and was propagated in antibiotic free medium.

Bulk cultures of these cells were used for differentiation and infection experiments in these studies. These iPSCs were washed with 2 ml PBS without calcium chloride and magnesium chloride. The cells were incubated with 1 ml of Gentle Cell Dissociation Reagent (Stemcell Technologies) for 6 min until the cells transformed into single cells. Single hiPSCs were collected and resuspended in FACS-PBS (PBS supplemented with 0.1% BSA and 0.1% sodium azide), counted and resuspended at 1 × 10^6^ cells/ml for use. Tubes containing 100,000 cells were incubated for 30 min at 4 °C with fluorochrome conjugated antibodies. Following incubation, cells were then washed once with PBS, removing any unbound antibodies and centrifuged at 1500 rpm for 5 min. Antibody binding to the surface of the cells was measured using the optimum concentration of an appropriate fluorochrome conjugated isotype specific antibody. In this study, unstained cells were used as a negative control. Measurement was carried out by using an electronic live gate on forward scatter and side scatter parameters. Data was acquired for 20,000–50,000 gated live events for each sample using a Novocyte flow cytometer (Agilent Technologies) equipped with a 488 nm laser and analysed using Novoexpress software.

### Formation of self-aggregated 3D hiPSCs spheroids

Agarose microplates were generated in 256-well format using the 3D Petri Dish® mould (Sigma Aldrich, Dorset) following the manufacturer instructions. These microplates were transferred to 12 well plates (Corning, Germany) as previously described [29, 48]. hiPSCs were expanded on laminin coated plates, were incubated with 1 ml of Gentle Dissociation Buffer (Stemcell Technologies) for 7–10 min at 37 °C. The single cell suspension was centrifuged at 0.2 rcf for 5 min and resuspended in mTeSR™1 supplemented with 10 µM Y-27,632 (Calbiochem, Watford) at a density of 2.0 × 106 live cells/ml. The prewarmed agarose microplates were seeded by transferring 190 ul of resulted cell suspension. After 2 h, 1 ml mTeSR™1 supplemented with 10 uM Y-27,632 was gently added to each well of 12-well plate and incubated overnight at 37 C.

### Hepatic induction of self-aggregated hiPSCs spheroids

Differentiation to HLC was performed as previously described [48, 49]. Differentiation was initiated by replacing mTeSR™1 with endoderm differentiation medium: RPMI1640 containing 1x B27 (Life Technologies), 100 ng/ml Activin A (PeproTech, Hammersmith), and 50 ng/ml Wnt3a (R&D Systems, Abingdon). The medium was changed every 24 h, for 72 h. On day 5, endoderm differentiation was substituted with hepatoblast differentiation medium. This medium was changed every second day for a further 5 days. This medium was composed of knockout-DMEM (Life Technologies), knockout serum replacement (KOSR-Life Technologies), 0.5% Glutamax (Life Technologies), 1% non-essential amino acids (Life-Technologies), 0.2% b-mercaptoethanol (Life Technologies), and 1% DMSO (Sigma-Aldrich). On day 10, hepatoblast medium was replaced with hepatocyte maturation medium HepatoZYME (Life Technologies) containing 1% Glutamax (Life-Technologies), supplemented with 10 ng/ml hepatocyte growth factor (HGF, PeproTech) and 20 ng /ml oncostatin M (OSM, PeproTech) as described previously [48, 49]. On day 21 of differentiation, cells were cultured in maintenance medium containing William’s E media (Life Technologies), supplemented with 10 ng/ml EGF (R&D systems), 10 ng/ml VEGF (R&D Systems), 10 ng/ml HGF (PeproTech), 10 ng/ml bFGF (PeproTech), 10% KOSR, 1% Glutamax, and 1% penicillin-streptomycin (Thermo Fisher Scientific) for the remining study, as previously described [49].

### Histology and immunofluorescence of 3D hepatospheres

3D spheroids were fixed in ice-cold methanol for 1 h, washed in PBS and embedded in agarose. Agarose-embedded spheroids were embedded in paraffin and 4 um sections were prepared. Antigen retrieval was performed using 1 x Tris-EDTA buffer solution for 15 min. Paraffin-embedded sections were also stained with Eosin and Hematoxylin and mounted in Pertex before microscopy. Brightfield images were taken using a Nikon Eclipse e600 microscope equipped with a Retiga 2000R camera (Q-imaging) and Image-Pro Premier software.

To stain sectioned hepatospheres, tissue was blocked with 10% BSA in PBS-tween (PBST) and incubated with primary antibody overnight at 4 C. Species-specific fluorescent-conjugated secondary antibody were used (Alexa Flour 488/Alexa Flour 568; Invitrogen). Sections were counterstained with DAPI (4’6-diamidino-2-phenylin-dole) and mounted with Fluoromount-G (SouthernBiotech) before microscopy.

### qPCR

RNA was extracted from iPSCs or 3D hepatospheres using RNAeasy Mini RNA Extraction Kit (Qiagen) according to manufacturer’s instructions. RNA quantity and quality were evaluated using Nanodrop^TM^200c. Following this step, cDNA was amplified using the RT2 First Strand Kit (Qiagen) following the manufacturer’s instruction. qPCR was performed with TaqMan Fast Advance Mastermix and primer pairs, using a Roche LightCycler 480 real-time PCR system. Gene expression was normalised to housekeeping gene; glyceraldehyde 3-phosphate dehydrogenase (GAPDH) and expressed as relative expression over 3D hepatospheres on day 0 of differentiation as control sample. qPCR experiments were conducted in triplicate and data was analysed using Roche LightCycler 480 software. Experiments were repeated with three biological repeats, infected with vector produced from the same production batch, with each samples run in triplicate.

### Hepatocyte phenotyping

To evaluate Cyp3A activity, 50 uM of Luciferin-PFBE substrate (Promega, Southampton) was incubated with 3D hepatospheres in HepatoZYME medium supplemented with 10 ng/ml HGF. Cytochrome P450 activity was measured 24 h later using the P450-Glo assay kit (Promega) following manufacturer’s instruction.

To measure AFP and ALB secretion, the supernatant was collected after 24 h and quantified using commercial ELISA kits (Alpha Diagnostics International, Texas). Data were normalised with the total protein content measured using bicinchoninic acid (BCA) assay (Thermo Fisher Scientific).

### Generation of high titre LV vectors

HEK293T cells were grown in DMEM GlutaMAX supplemented with 10% foetal bovine serum and 1% Penicillin Streptomycin (Fisher Scientific, Loughborough), at 37 °C with 5% CO_2_. Cells were passaged regularly upon confluency.

pHR’SIN-cPPT-SEW (pHR) and it’s native LTR counterpart (pHV) LV were generated as previously described [50]. These vectors are used reference standards, carrying *eGFP* under SFFV promoter regulation and are identical apart from the LTR configuration (Fig. S1A). Briefly, 1.5 × 10^7^ HEK293T cells were seeded per T175 flask and incubating at 37 °C, 5% CO_2_ overnight. Cells were transfected with 16 µg eGFP transgene, 12 µg pCMVR8.74 and 4 µg pMD2.G with a transfection reagent in serum free medium. Medium was replaced 24 h post transfection and supernatant harvested every 24 h for 72 h post replacement. Conditioned medium was filtered through 0.45 µM filters (Fisher Scientific) and stored at 4 °C for future use.

Conditioned medium was concentrated via ultracentrifugation at 23,000 rpm at 4 °C for 2 ½ h, using an SW32Ti rotor and Optima XPN ultracentrifuge (Beckman Coulter, High Wycombe). Viral pellet was resuspended in 200 µl serum free medium and stored at −80 °C for future use.

### LV titration

Infectious LV titre was calculated by as previously reported [50]. Briefly, 2 × 105 HEK293T cells were seeded and incubated at 37 °C, 5% CO_2_ overnight to adhere. Serial dilutions of concentrated LV were prepared and incubated in complete cell culture medium with 5 µg/ml polybrene (Sigma-Aldrich), for 20 min at room temperature before addition to cells. Medium was replaced after 24 h incubation and incubated for a further 48 h before analysis using a Novocyte flow cytometer (ACEA Biosciences Inc, San Diego) and data analysis using NovoExpress software. Dilutions expressing 1–30% GFP expression were analysed as accurate representations of viral titre (TU/ml), calculated as below:

$$(({{{\rm{Cell}}}}\; {{{\rm{count}}}}\times ({{{\rm{Percentage}}}}\; {{{\rm{GFP}}}}\; {{{\rm{expression}}}}/100))/{{{\rm{Volume}}}})* {{{\rm{Dilution}}}}\; {{{\rm{factor}}}}$$

### Optimisation of iPSC and HLC gene transfer

One day prior to transduction, 3 × 105 iPSCs were seeded in pre-coated laminin plates. The following days, one well of the cells were dissociated using Gentle Cell Dissociation reagent (StemCell Technology). For lentiviral transduction, mTeSR1 medium containing 10 µM Y-27,632 (Calbiochem) and 5 µg/ml polybrene reagent (Sigma-Aldrich, UK) was prepared. The virus added to the medium and the mixture was incubated for 20 min at room temperature. The medium from the cells removed and replaced with the medium containing the virus and rock inhibitor. The plate was incubated at 37 °C for 24 h. Following day, the transduction medium was replaced with fresh complete mTesr1 medium. This step was continued for 3 days and medium refreshed daily. The cells were extracted for flow cytometry analysis to determine the number of GFP positive cells.

For 3D hepatospheres, William’s E medium (Life Technology) supplemented with 10 µM Y-27,632, 10 ng/ml EGF (R&D Systems), 10 ng/ml VEGF (R&D Systems), 10 ng/ml HGF (PeproTech), 10 ng/ml bFGF (PeproTech), 5 µg/ml polybrene (Sigma-Aldrich) and the virus was prepared. The mixture was incubated for 20 min at room temperature before adding to the cells. The transduction medium added to the cells and incubated for 24 h before with complete William’s E medium with essential growth factor supplements. Following transduction, transduced 3D hepatocytes were kept 3 to 7 days until fluorescent cells appeared.

iPS cells were harvested for analysis 3 days and 30 days after infection using pHR or pHV LV and HLCs were only analysed at day 3 post infection. Three biological replicates were used per samples.

### Cloning iPSC

Prior to single cell cloning, iPSCs were transduced with lentiviral vectors as previously described. On day of single cell cloning complete mTeSR1 (StemCell Technology) medium with conditioned medium at a ratio of 1:1 was prepared. The medium was supplemented with 10 µM Y-27,632 (Calbiochem) to enhance the cell survival. Transduced positive GFP cells were washed with PBS (Sigma-Aldrich) once and gently dissociated using Gentle Cell Dissociation reagent (StemCell Technology) for 15 min. The single cells were resuspended in mTeSR1 and Y-2763 and counted by haemocytometer. A 2 cells/ml solution was generated by dilution with complete/conditioned medium and 500 µl seeded in a laminin-521 coated 24-well plate. This was to ensure the plate was seeded at a density of 1 cell/well. Following seeding, the cells were undisturbed for 7 to 10 days. After 7 days, the plate was scanned for colonies. The cells from each colony were expanded and harvested for DNA/RNA extraction. Thirty-three (pHR LV) and 7 (pHV LV) clones were isolated from infected samples respectively.

### Nucleic acid isolation

DNA and RNA samples were isolated from transduced samples according using DNeasy Blood & Tissue Kit and RNeasy Mini Kit (Qiagen, Manchester) respectively, according to manufacturer’s instructions (*n* = 3). RNA was treated with DNAase I to remove contaminants, according to manufactures instructions (Qiagen). Nucleic acid concentration and purity was analysed using NanoDrop™ 2000c spectrophotometer (Thermo Fisher Scientific, Hemel Hempstead).

### Nucleic acid integrity analysis

DNA samples were initially analysed for vector presence (*n* = 3). Therefore, a vector specific primer pair was designed and used in a standard PCR with 10 ng DNA. Products were applied to 2% gel electrophoresis to visualise PCR amplicons and the detection of expected bands.

### RNAseq sample preparation for fusion transcript and expression analysis

RNA quality was assessed on TapeStation 2200 system using TapeStation RNA ScreenTape & Reagents (Agilent, Santa Clara). Up to 1000 ng total RNA per sample were applied to SureSelect Strand-Specific mRNA Library Preparation for Illumina (Agilent) and TruSeq Stranded mRNA Library Prep Kit (Illumina) according to manufacturer’s instructions for library preparation. Libraries were sequenced in 150PE mode on Illumina HiSeq System. Sequencing data was analysed using GENE-IS [51] for the detection of fusion transcripts and DEseq2 for the assessment of differentially expressed genes.

### Vector copy number analysis

Vector copy number in the samples were determined by quantitative TaqMan™ Universal PCR Master Mix. Briefly, 10 ng of DNA were applied in triplicate analysis on CFX96 Touch Real-Time PCR Detection System (Bio-Rad, Hercules). A standard curve of 8 calibration standards (10^7^ – 5 copies) was generated for absolute quantification of vector copies in the samples. LV specific primers and probes were designed and ordered from Sigma-Aldrich (Munich, Germany) and IDT Technologies (Coralville, USA), respectively. Primers were used at a final concentration of 720 nM, probes at 140 nM in a total reaction volume of 20 µl.

### Analysis of vector integration sites

Viral vector integration sites analysis was performed using S-EPTS/LM-PCR, which is a shearing DNA based integration site analysis method, followed by next-generation sequencing.

Raw sequencing data were trimmed based on quality (Phred) and filtered for containing both molecular barcodes at full identity. Remaining reads were further analysed using GENE-IS. Briefly, sequences were further trimmed and only sequences containing the expected vector-specific stretch were considered for following steps. First, sequences were aligned to the human genome (UCSC assembly release number hg38) by Burrows-Wheeler Aligner (BWA) MEM algorithm. Potential integration sites were then mapped with BLAST at a minimum alignment identity percentage of 95%. Adjacent genes and other features were annotated according to RefSeq database. For each detected integration site, the relative sequence count compared to all sequences was calculated.

Count matrix data was normalised to quantify the proportion of gene with viral insertions.

Statistical significance was determined using the Student’s *t* test, with *p* values adjusted using the Benjamini–Hochberg method. Comparisons were made between iPSC early (day 3) and late (day 30) IS for each genomic region (exon, intron, 3’ UTR and 5’ UTR) between samples infected with pHR or pHV LV. For HLC samples, data was compared between pHR and pHV LV infected samples.

### Analysis of common integration sites

Biologically relevant IS clusters, called common integration sites (CIS), were analysed using a graphs based approach. Any IS detected was considered as node that contained the IS locus. If the distance of two nodes was less than 50 kb, the nodes were connected and resulting nodes sets considered as CIS.

### Integration sites in proximity to cancer-related genes

A list of over 700 well-defined cancer genes was compiled from the Cancer Gene Census database (https://cancer.sanger.ac.uk/census). Cancer gene data was obtained from Ensembl human genes (http://www.ensembl.org/biomart/martview/; version GRCh38.p10). Relative frequencies of integration sites, that were detected within a 100 kb window of a TSS of a cancer-related gene, were analysed.

### Methylome analysis using the Illumina Epic Kit

Up to 250 ng DNA per sample were applied to Infinium Methylation EPIC Kit (Illumina, San Diego) according to manufacturer’s instructions and arrays were scanned on Illumina’s iScan System. Generated data was analysed using ‘The Chip Analysis Methylation Pipeline’ in order to determine differentially methylated regions.

### Bioinformatic analysis pipeline

We established an analytical pipeline in R (v.4.0.2). Raw data was curated into feature-sample matrices and streamlined into ExpressionSet objects using BioBase (v.2.50.0). The subsequent analysis mainly includes differential analysis, gene set enrichment analysis, signature score assessment, and weighted gene co-expression network analysis. Gene enrichment analysis were performed using the hypergeometric method, and gene classes analysed with a Benjamini– Hochberg adjusted *p* value of less than 0.05, using

#### Differential analysis

We used limma (v3.46.0) for differential analysis on count matrices. First, we normalised and conducted log2-transformation on count matrices. Next, we constructed design matrices using phenotypic data and fitted these to the processed matrices. Following empirical Bayes moderation, we identified differentially expressed genes (DEGs) and retained those with an absolute log2 fold-change greater than 1 and BH-adjusted *p* values less than 0.01. Lastly, we visualised these DEGs using EnhancedVolcano (v.1.8.0).

#### Gene Set Enrichment Analysis (GSEA)

We used clusterProfiler (v3.18.1) for GSEA. First, we ranked gene expression levels from highest to lowest by comparing transcriptomic samples from study groups against controls. Next, we used Hallmark gene sets or GO terms from MSigDB (v.7.5.1) to perform GSEA, resulting in BH-adjusted *p* values, ratio of genes from each set, and other attributes. Lastly, we used treeplot or cnetplot to visualise the results.

#### Signature score analysis

We defined molecular signatures for specific cancers using TCGA RNA-seq datasets. Differential analysis criteria for these signature genes were set at log2 fold-change >2 and BH-adjusted *p* values < 0.01. We then determined the scaled mean expression levels of these genes and visualised them using pheatmap (v1.0.12).

#### Weighted gene co-expression network analysis (WGCNA)

We used WGCNA (v.1.70-3) for the analysis. We began with normalised and transposed count matrices and performed network topology analysis, resulting in an optimal soft threshold power. We then constructed topological overlap matrix using the blockwiseModules command and determine traits associated with each module by calculating hypothetical central genes. Lastly, we identified potential key drivers in selected modules by using the intramodularConnectivity command.

## Supplementary information

Supplementary figure S1

Supplementary figure S2 A-D

Supplementary figure S3

Supplementary figure S4

Supplementary figure S5. Pathway analysis showing enriched GO terms or KEGG across different samples.

Supplementary table S1. Differential sequence count chances in oncogene and tumour suppressor gene IS across iPSC samples over 30 days

Supplementary table S2. Omics analysis identifying IS and fusion gene transcripts revealing overlap between genotoxicity outreads

Supplementary table S3. Co-expression modules reveal key gene subsets with diverse functional implications across infected iPSCs and HLCs.

## Data Availability

Raw data are available upon reasonable request to the corresponding author.
