# Supplementary material for: hInGeTox: a human-based in vitro platform to evaluate lentivirus/host interactions that contribute to genotoxicity
Source: Gene Ther. 2025 Jul 15;32(6):641–56. doi: 10.1038/s41434-025-00550-9 (PMC12714580; doi:10.1038/s41434-025-00550-9)
Supplement: Supplementary file 1 — Supplementary figure S1 [file 41434_2025_550_MOESM1_ESM.pptx]

## Slide 1
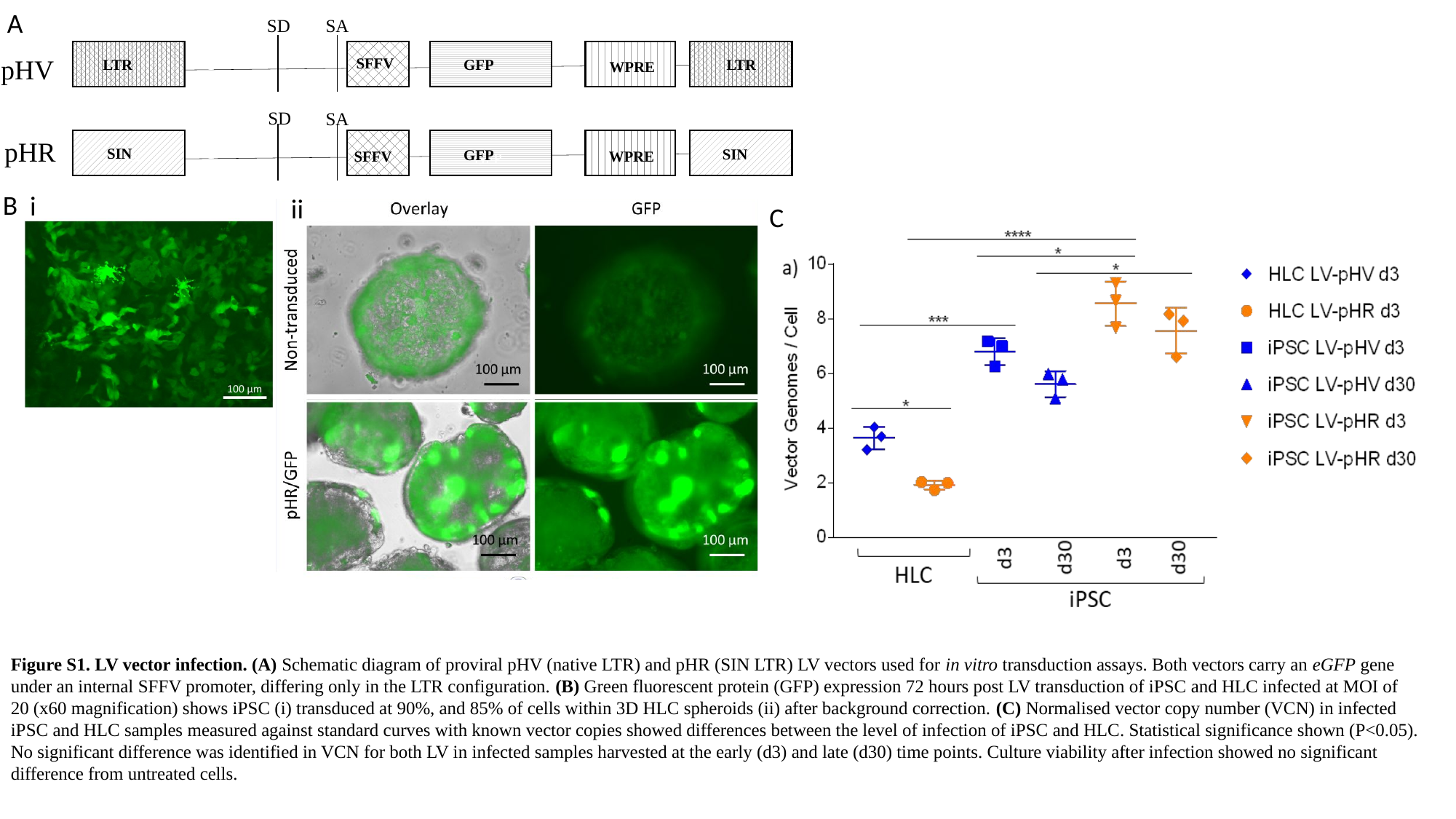

A
SA
SD
pHV
Y
SFFV
LTR
GFP
LTR
WPRE
SD
SA
pHR
Y
SIN
SIN
GFP
SFFV
WPRE
SIN
GFP
B
i
ii
C
Figure S1. LV vector infection. (A) Schematic diagram of proviral pHV (native LTR) and pHR (SIN LTR) LV vectors used for in vitro transduction assays. Both vectors carry an eGFP gene under an internal SFFV promoter, differing only in the LTR configuration. (B) Green fluorescent protein (GFP) expression 72 hours post LV transduction of iPSC and HLC infected at MOI of 20 (x60 magnification) shows iPSC (i) transduced at 90%, and 85% of cells within 3D HLC spheroids (ii) after background correction. (C) Normalised vector copy number (VCN) in infected iPSC and HLC samples measured against standard curves with known vector copies showed differences between the level of infection of iPSC and HLC. Statistical significance shown (P<0.05). No significant difference was identified in VCN for both LV in infected samples harvested at the early (d3) and late (d30) time points. Culture viability after infection showed no significant difference from untreated cells.
