# Supplementary material for: hInGeTox: a human-based in vitro platform to evaluate lentivirus/host interactions that contribute to genotoxicity
Source: Gene Ther. 2025 Jul 15;32(6):641–56. doi: 10.1038/s41434-025-00550-9 (PMC12714580; doi:10.1038/s41434-025-00550-9)
Supplement: Supplementary file 2 — Supplementary figure S2 A-D [file 41434_2025_550_MOESM2_ESM.pptx]

## Slide 1
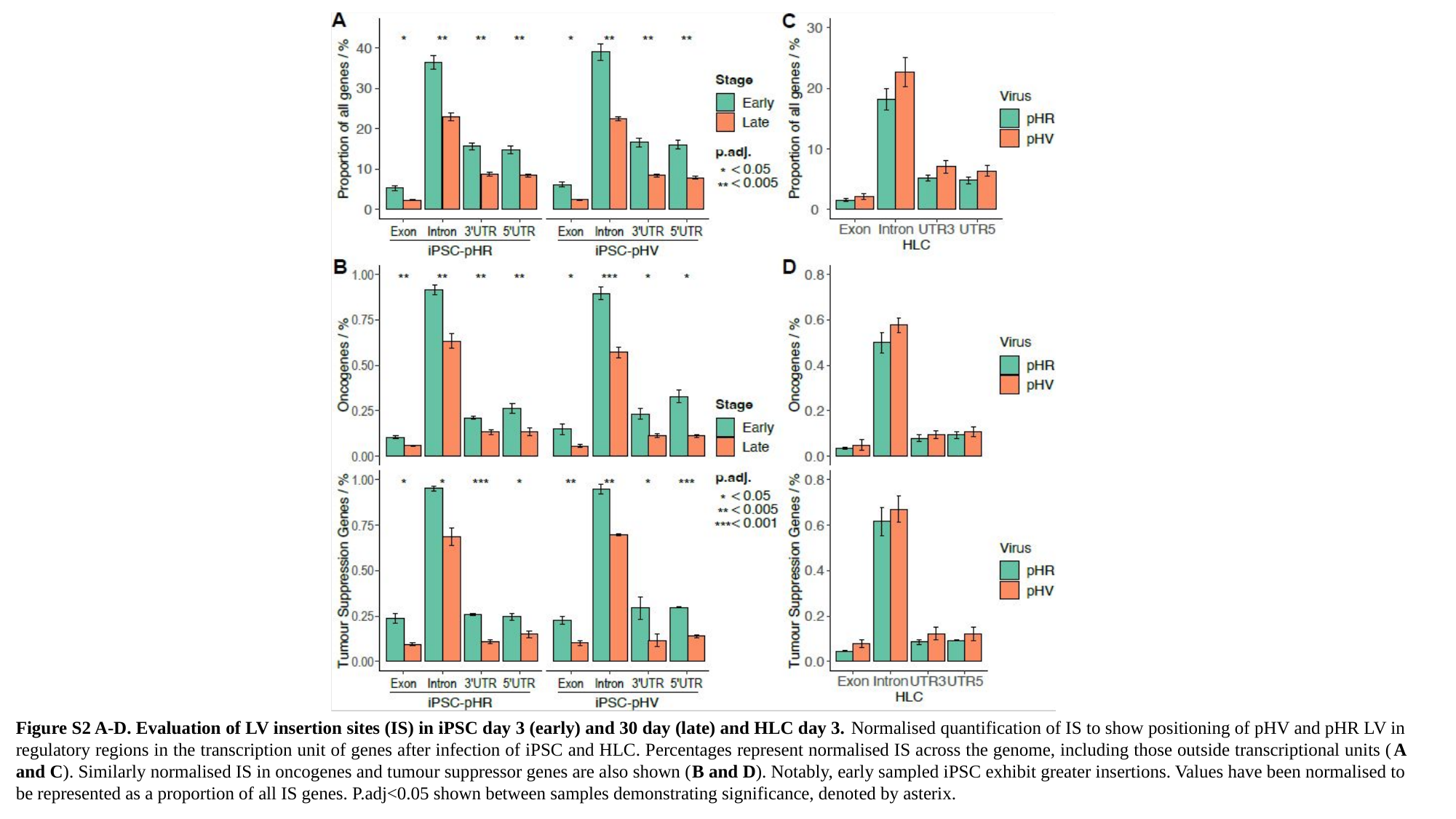

Figure S2 A-D. Evaluation of LV insertion sites (IS) in iPSC day 3 (early) and 30 day (late) and HLC day 3. Normalised quantification of IS to show positioning of pHV and pHR LV in regulatory regions in the transcription unit of genes after infection of iPSC and HLC. Percentages represent normalised IS across the genome, including those outside transcriptional units (A and C). Similarly normalised IS in oncogenes and tumour suppressor genes are also shown (B and D). Notably, early sampled iPSC exhibit greater insertions. Values have been normalised to be represented as a proportion of all IS genes. P.adj<0.05 shown between samples demonstrating significance, denoted by asterix.
