# Supplementary material for: hInGeTox: a human-based in vitro platform to evaluate lentivirus/host interactions that contribute to genotoxicity
Source: Gene Ther. 2025 Jul 15;32(6):641–56. doi: 10.1038/s41434-025-00550-9 (PMC12714580; doi:10.1038/s41434-025-00550-9)
Supplement: Supplementary file 3 — Supplementary figure S3 [file 41434_2025_550_MOESM3_ESM.pptx]

## Slide 1
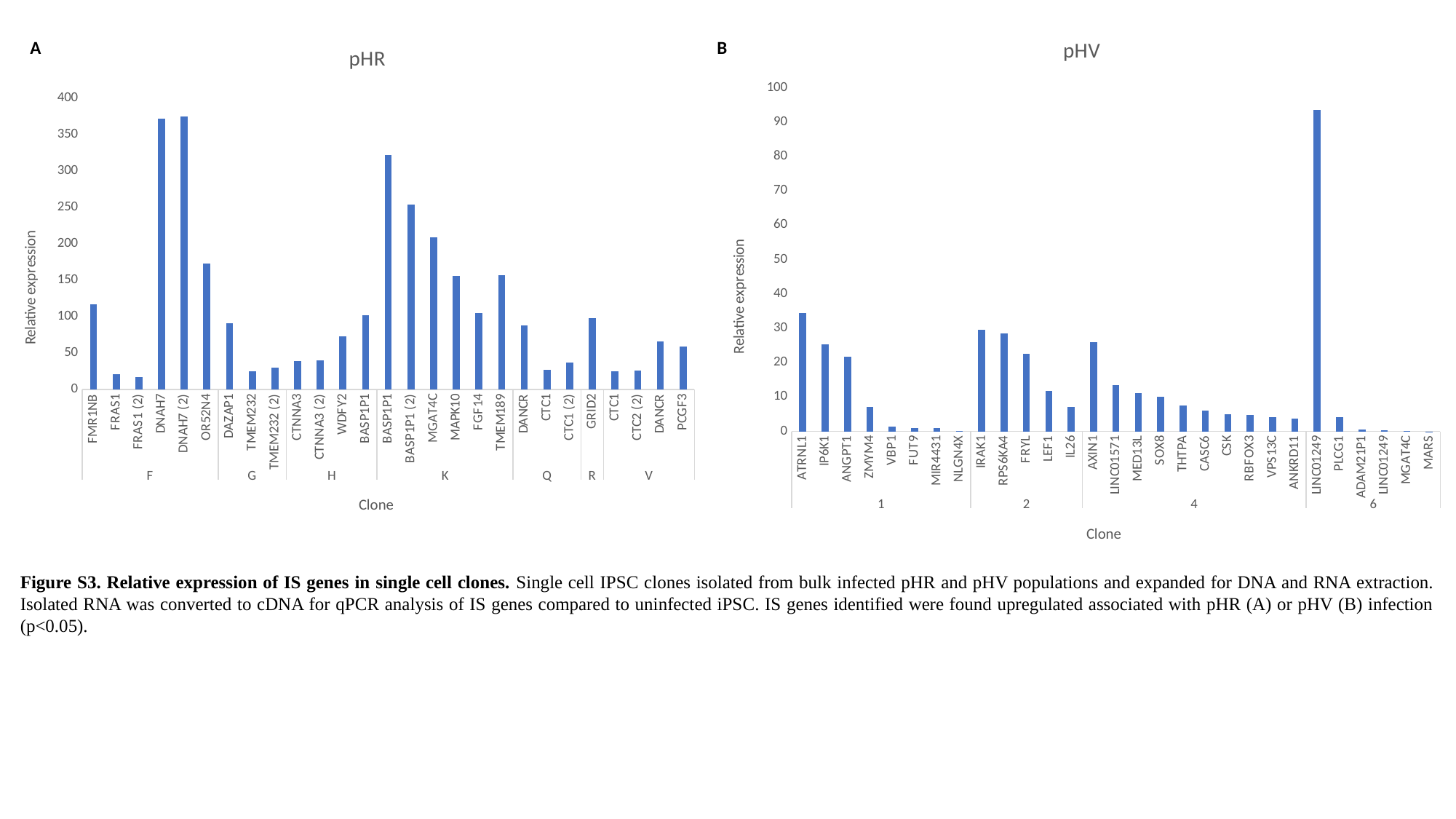

### Chart: pHV
| Category | |
|---|---|
| ATRNL1 | 34.452 |
| IP6K1 | 25.284 |
| ANGPT1 | 21.836 |
| ZMYM4 | 7.199 |
| VBP1 | 1.314 |
| FUT9 | 0.98 |
| MIR4431 | 0.947 |
| NLGN4X | 0.211 |
| IRAK1 | 29.472 |
| RPS6KA4 | 28.589 |
| FRYL | 22.486 |
| LEF1 | 11.839 |
| IL26 | 7.11 |
| AXIN1 | 25.903 |
| LINC01571 | 13.498 |
| MED13L | 11.187 |
| SOX8 | 10.163 |
| THTPA | 7.512 |
| CASC6 | 6.114 |
| CSK | 5.008 |
| RBFOX3 | 4.818 |
| VPS13C | 4.152 |
| ANKRD11 | 3.65 |
| LINC01249 | 93.454 |
| PLCG1 | 4.119 |
| ADAM21P1 | 0.569 |
| LINC01249 | 0.319 |
| MGAT4C | 0.135 |
| MARS | 0.001 |
### Chart: pHR
| Category | |
|---|---|
| FMR1NB | 117.0 |
| FRAS1 | 21.0 |
| FRAS1 (2) | 17.0 |
| DNAH7 | 372.0 |
| DNAH7 (2) | 375.0 |
| OR52N4 | 173.0 |
| DAZAP1 | 91.0 |
| TMEM232 | 25.0 |
| TMEM232 (2) | 30.0 |
| CTNNA3 | 39.0 |
| CTNNA3 (2) | 40.0 |
| WDFY2 | 73.0 |
| BASP1P1 | 102.0 |
| BASP1P1 | 322.0 |
| BASP1P1 (2) | 254.0 |
| MGAT4C | 209.0 |
| MAPK10 | 156.0 |
| FGF14 | 105.0 |
| TMEM189 | 157.0 |
| DANCR | 88.0 |
| CTC1 | 27.0 |
| CTC1 (2) | 37.0 |
| GRID2 | 98.0 |
| CTC1 | 25.0 |
| CTC2 (2) | 26.0 |
| DANCR | 66.0 |
| PCGF3 | 59.0 |A
B
Figure S3. Relative expression of IS genes in single cell clones. Single cell IPSC clones isolated from bulk infected pHR and pHV populations and expanded for DNA and RNA extraction. Isolated RNA was converted to cDNA for qPCR analysis of IS genes compared to uninfected iPSC. IS genes identified were found upregulated associated with pHR (A) or pHV (B) infection (p<0.05).
