# Supplementary material for: hInGeTox: a human-based in vitro platform to evaluate lentivirus/host interactions that contribute to genotoxicity
Source: Gene Ther. 2025 Jul 15;32(6):641–56. doi: 10.1038/s41434-025-00550-9 (PMC12714580; doi:10.1038/s41434-025-00550-9)
Supplement: Supplementary file 4 — Supplementary figure S4 [file 41434_2025_550_MOESM4_ESM.pptx]

## Slide 1
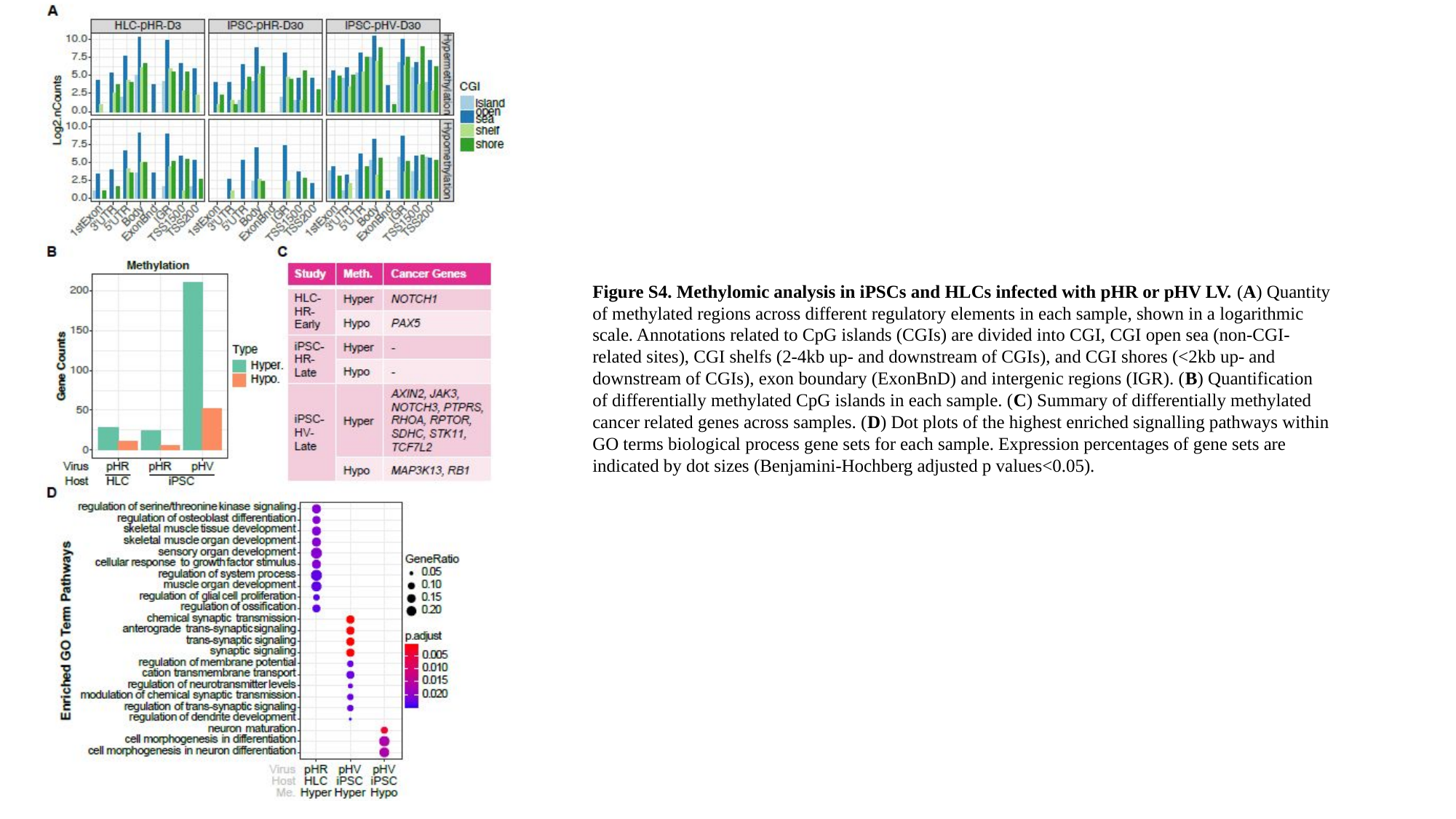

Figure S4. Methylomic analysis in iPSCs and HLCs infected with pHR or pHV LV. (A) Quantity of methylated regions across different regulatory elements in each sample, shown in a logarithmic scale. Annotations related to CpG islands (CGIs) are divided into CGI, CGI open sea (non-CGI-related sites), CGI shelfs (2-4kb up- and downstream of CGIs), and CGI shores (<2kb up- and downstream of CGIs), exon boundary (ExonBnD) and intergenic regions (IGR). (B) Quantification of differentially methylated CpG islands in each sample. (C) Summary of differentially methylated cancer related genes across samples. (D) Dot plots of the highest enriched signalling pathways within GO terms biological process gene sets for each sample. Expression percentages of gene sets are indicated by dot sizes (Benjamini-Hochberg adjusted p values<0.05).
