# Supplementary material for: hInGeTox: a human-based in vitro platform to evaluate lentivirus/host interactions that contribute to genotoxicity
Source: Gene Ther. 2025 Jul 15;32(6):641–56. doi: 10.1038/s41434-025-00550-9 (PMC12714580; doi:10.1038/s41434-025-00550-9)
Supplement: Supplementary file 5 — Supplementary figure S5. Pathway analysis showing enriched GO terms or KEGG across different samples. [file 41434_2025_550_MOESM5_ESM.pptx]

## Slide 1
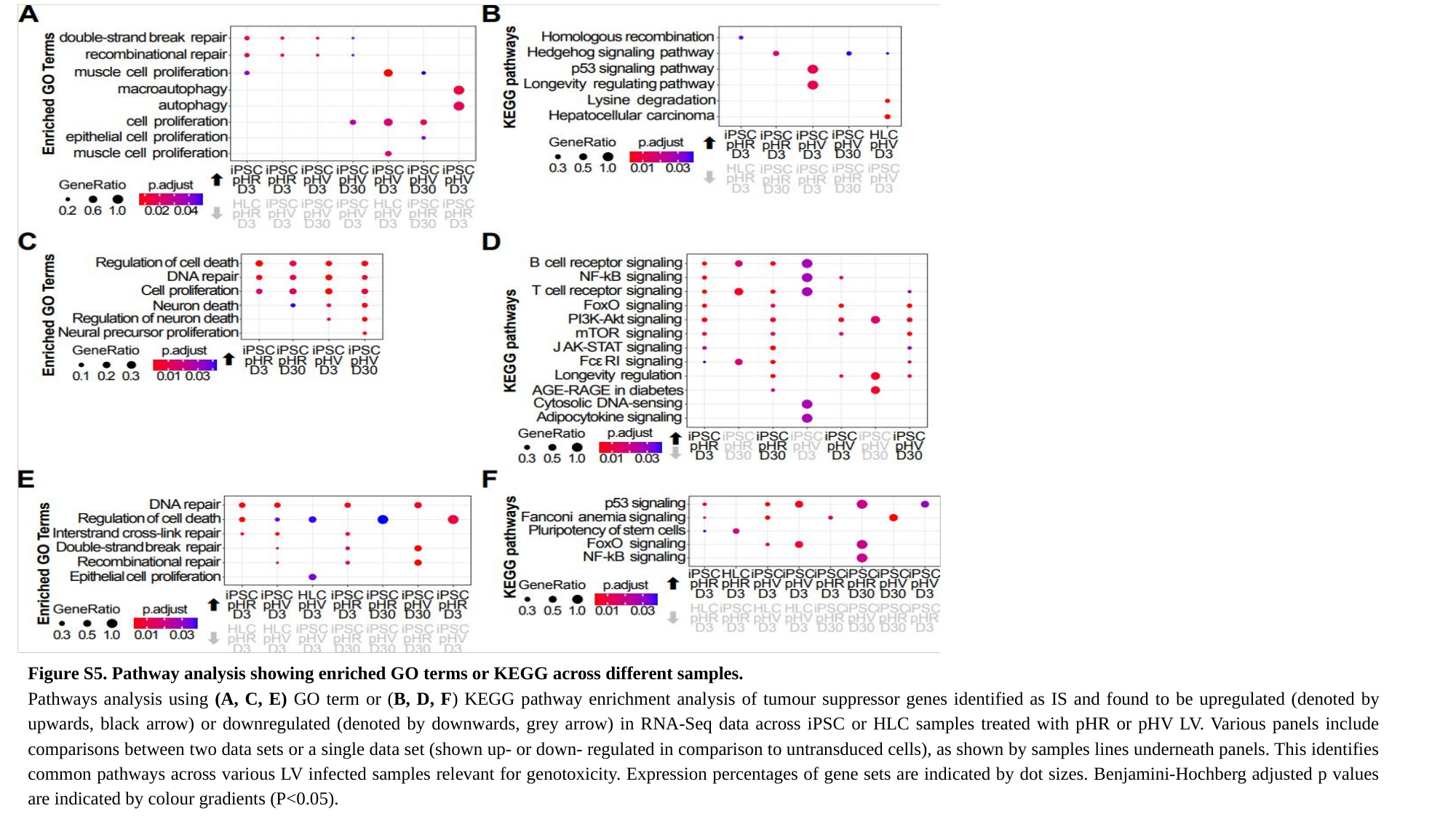

Figure S5. Pathway analysis showing enriched GO terms or KEGG across different samples.
Pathways analysis using (A, C, E) GO term or (B, D, F) KEGG pathway enrichment analysis of tumour suppressor genes identified as IS and found to be upregulated (denoted by upwards, black arrow) or downregulated (denoted by downwards, grey arrow) in RNA-Seq data across iPSC or HLC samples treated with pHR or pHV LV. Various panels include comparisons between two data sets or a single data set (shown up- or down- regulated in comparison to untransduced cells), as shown by samples lines underneath panels. This identifies common pathways across various LV infected samples relevant for genotoxicity. Expression percentages of gene sets are indicated by dot sizes. Benjamini-Hochberg adjusted p values are indicated by colour gradients (P<0.05).
