# Supplementary material for: hInGeTox: a human-based in vitro platform to evaluate lentivirus/host interactions that contribute to genotoxicity
Source: Gene Ther. 2025 Jul 15;32(6):641–56. doi: 10.1038/s41434-025-00550-9 (PMC12714580; doi:10.1038/s41434-025-00550-9)
Supplement: Supplementary file 6 — Supplementary table S1. Differential sequence count chances in oncogene and tumour suppressor gene IS across iPSC samples over 30 days [file 41434_2025_550_MOESM6_ESM.pptx]

## Slide 1
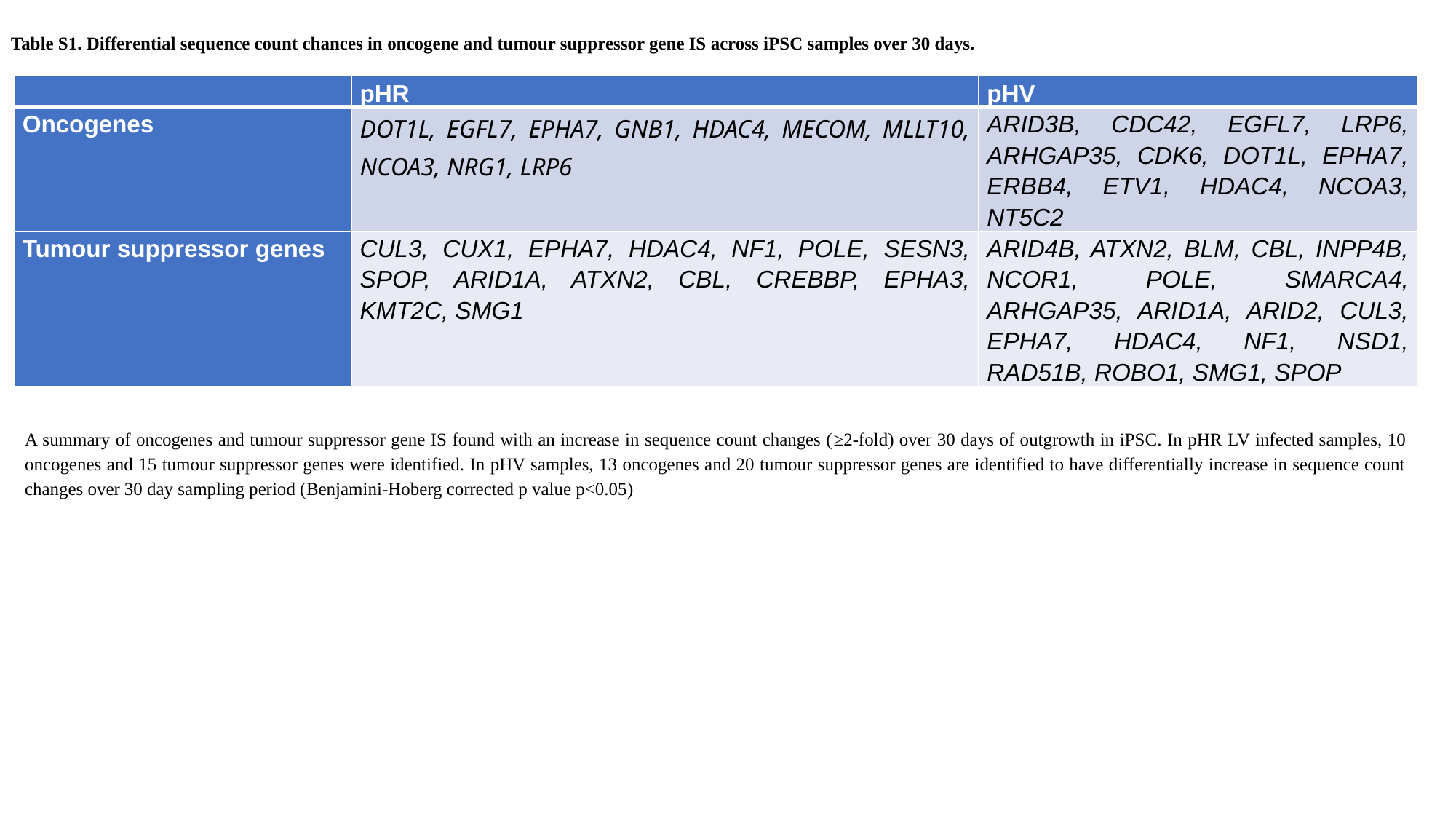

Table S1. Differential sequence count chances in oncogene and tumour suppressor gene IS across iPSC samples over 30 days.
| | pHR | pHV |
| --- | --- | --- |
| Oncogenes | DOT1L, EGFL7, EPHA7, GNB1, HDAC4, MECOM, MLLT10, NCOA3, NRG1, LRP6 | ARID3B, CDC42, EGFL7, LRP6, ARHGAP35, CDK6, DOT1L, EPHA7, ERBB4, ETV1, HDAC4, NCOA3, NT5C2 |
| Tumour suppressor genes | CUL3, CUX1, EPHA7, HDAC4, NF1, POLE, SESN3, SPOP, ARID1A, ATXN2, CBL, CREBBP, EPHA3, KMT2C, SMG1 | ARID4B, ATXN2, BLM, CBL, INPP4B, NCOR1, POLE, SMARCA4, ARHGAP35, ARID1A, ARID2, CUL3, EPHA7, HDAC4, NF1, NSD1, RAD51B, ROBO1, SMG1, SPOP |
A summary of oncogenes and tumour suppressor gene IS found with an increase in sequence count changes (≥2-fold) over 30 days of outgrowth in iPSC. In pHR LV infected samples, 10 oncogenes and 15 tumour suppressor genes were identified. In pHV samples, 13 oncogenes and 20 tumour suppressor genes are identified to have differentially increase in sequence count changes over 30 day sampling period (Benjamini-Hoberg corrected p value p<0.05)
