# Supplementary material for: hInGeTox: a human-based in vitro platform to evaluate lentivirus/host interactions that contribute to genotoxicity
Source: Gene Ther. 2025 Jul 15;32(6):641–56. doi: 10.1038/s41434-025-00550-9 (PMC12714580; doi:10.1038/s41434-025-00550-9)
Supplement: Supplementary file 7 — Supplementary table S2. Omics analysis identifying IS and fusion gene transcripts revealing overlap between genotoxicity outreads [file 41434_2025_550_MOESM7_ESM.pptx]

## Slide 1
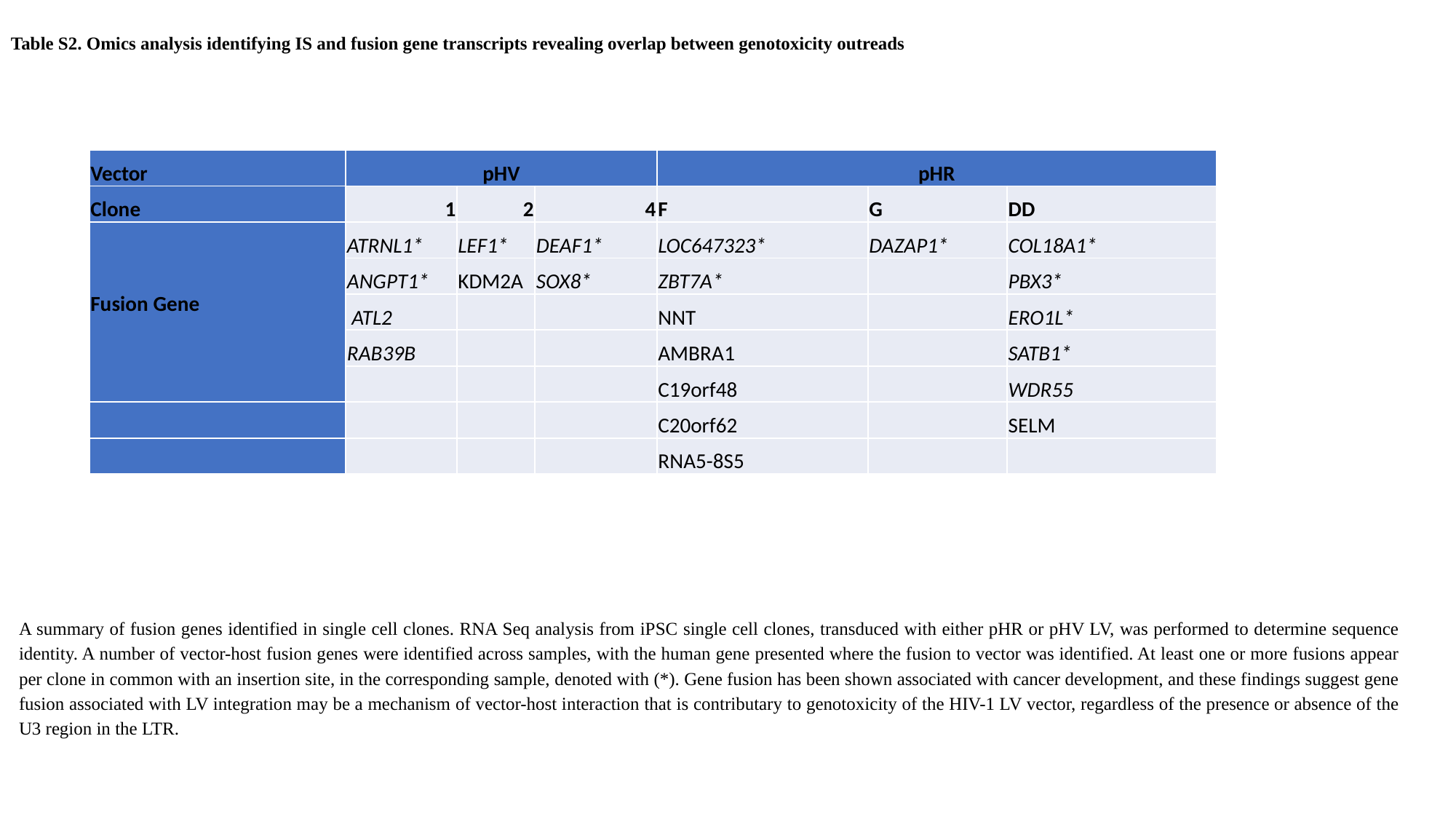

Table S2. Omics analysis identifying IS and fusion gene transcripts revealing overlap between genotoxicity outreads
| Vector | pHV | | | pHR | | |
| --- | --- | --- | --- | --- | --- | --- |
| Clone | 1 | 2 | 4 | F | G | DD |
| Fusion Gene | ATRNL1\* | LEF1\* | DEAF1\* | LOC647323\* | DAZAP1\* | COL18A1\* |
| | ANGPT1\* | KDM2A | SOX8\* | ZBT7A\* | | PBX3\* |
| | ATL2 | | | NNT | | ERO1L\* |
| | RAB39B | | | AMBRA1 | | SATB1\* |
| | | | | C19orf48 | | WDR55 |
| | | | | C20orf62 | | SELM |
| | | | | RNA5-8S5 | | |
A summary of fusion genes identified in single cell clones. RNA Seq analysis from iPSC single cell clones, transduced with either pHR or pHV LV, was performed to determine sequence identity. A number of vector-host fusion genes were identified across samples, with the human gene presented where the fusion to vector was identified. At least one or more fusions appear per clone in common with an insertion site, in the corresponding sample, denoted with (*). Gene fusion has been shown associated with cancer development, and these findings suggest gene fusion associated with LV integration may be a mechanism of vector-host interaction that is contributary to genotoxicity of the HIV-1 LV vector, regardless of the presence or absence of the U3 region in the LTR.
