# Supplementary material for: hInGeTox: a human-based in vitro platform to evaluate lentivirus/host interactions that contribute to genotoxicity
Source: Gene Ther. 2025 Jul 15;32(6):641–56. doi: 10.1038/s41434-025-00550-9 (PMC12714580; doi:10.1038/s41434-025-00550-9)
Supplement: Supplementary file 8 — Supplementary table S3. Co-expression modules reveal key gene subsets with diverse functional implications across infected iPSCs and HLCs. [file 41434_2025_550_MOESM8_ESM.pptx]

## Slide 1
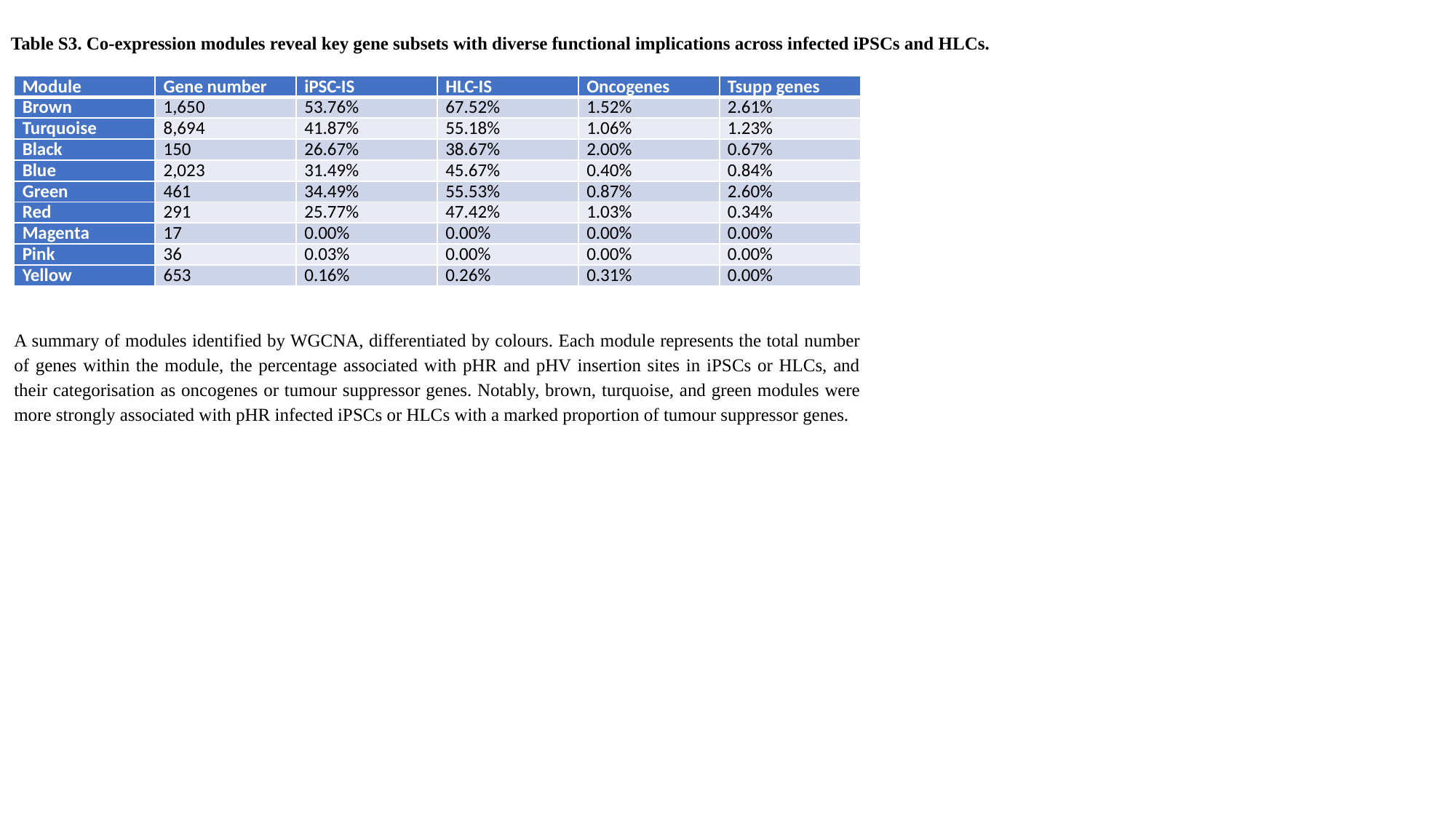

Table S3. Co-expression modules reveal key gene subsets with diverse functional implications across infected iPSCs and HLCs.
| Module | Gene number | iPSC-IS | HLC-IS | Oncogenes | Tsupp genes |
| --- | --- | --- | --- | --- | --- |
| Brown | 1,650 | 53.76% | 67.52% | 1.52% | 2.61% |
| Turquoise | 8,694 | 41.87% | 55.18% | 1.06% | 1.23% |
| Black | 150 | 26.67% | 38.67% | 2.00% | 0.67% |
| Blue | 2,023 | 31.49% | 45.67% | 0.40% | 0.84% |
| Green | 461 | 34.49% | 55.53% | 0.87% | 2.60% |
| Red | 291 | 25.77% | 47.42% | 1.03% | 0.34% |
| Magenta | 17 | 0.00% | 0.00% | 0.00% | 0.00% |
| Pink | 36 | 0.03% | 0.00% | 0.00% | 0.00% |
| Yellow | 653 | 0.16% | 0.26% | 0.31% | 0.00% |
A summary of modules identified by WGCNA, differentiated by colours. Each module represents the total number of genes within the module, the percentage associated with pHR and pHV insertion sites in iPSCs or HLCs, and their categorisation as oncogenes or tumour suppressor genes. Notably, brown, turquoise, and green modules were more strongly associated with pHR infected iPSCs or HLCs with a marked proportion of tumour suppressor genes.
